# MCP5, a methyl-accepting chemotaxis protein regulated by both the Hk1-Rrp1 and Rrp2-RpoN-RpoS pathways, is required for the immune evasion of *Borrelia burgdorferi*

**DOI:** 10.1371/journal.ppat.1012327

**Published:** 2024-12-30

**Authors:** Sajith Raghunandanan, Kai Zhang, Yan Zhang, Raj Priya, Ching Wooen Sze, Yongliang Lou, Michael J. Lynch, Brian R. Crane, Mark H. Kaplan, Chunhao Li, X. Frank Yang

**Affiliations:** 1 Department of Microbiology and Immunology, Indiana University School of Medicine, Indianapolis, Indiana, United States of America; 2 Department of Oral Craniofacial Molecular Biology, School of Dentistry, Virginia Commonwealth University, Richmond, Virginia, United States of America; 3 Wenzhou Key Laboratory of Sanitary Microbiology, Key Laboratory of Laboratory Medicine, Ministry of Education, School of Laboratory Medicine and Life Sciences, Wenzhou Medical University, Wenzhou, China; 4 Department of Chemistry and Chemical Biology, Cornell University, Ithaca, New York, United States of America; Tufts Univ School of Medicine, UNITED STATES OF AMERICA

## Abstract

*Borrelia* (or *Borreliella*) *burgdorferi*, the causative agent of Lyme disease, is a motile and invasive zoonotic pathogen adept at navigating between its arthropod vector and mammalian host. While motility and chemotaxis are well known to be essential for its enzootic cycle, the role of each methyl-accepting chemotaxis proteins (MCPs) in the infectious cycle of *B*. *burgdorferi* remains unclear. In this study, we show that *mcp5*, a gene encoding one of the most abundant MCPs in *B*. *burgdorferi*, is differentially expressed in response to environmental signals and at distinct stages of the pathogen’s enzootic cycle. Notably, *mcp5* expression is regulated by the Hk1-Rrp1 and Rrp2-RpoN-RpoS pathways, two key regulatory pathways that are critical for the spirochete’s colonization of the tick vector and mammalian host, respectively. Infection experiments with an *mcp5* mutant revealed that spirochetes lacking MCP5 were unable to establish infections in either C3H/HeN mice or Severe Combined Immunodeficiency (SCID) mice, which are deficient in adaptive immunity, underscoring MCP5’s critical role in mammalian infection. However, the *mcp5* mutant was able to establish infection and disseminate in NOD SCID Gamma (NSG) mice, which are deficient in both adaptive and most innate immune responses, suggesting that MCP5 plays an important role in evading host innate immunity. Moreover, NK cell depletion in C3H and SCID mice restored the infectivity of the *mcp5* mutant, further highlighting MCP5’s role in evading NK cell-associated immunity. Co-culture assays with NK cells and macrophages revealed that the *mcp5* mutant enhanced interferon-gamma production by NK cells. In the tick vector, the *mcp5* mutants survived feeding but failed to transmit to mice. These findings reveal that MCP5, regulated by both the Rrp1 and Rrp2 pathways, is critical for establishing infection in mammalian hosts by evading NK cell-mediated host innate immunity and is important for the transmission of spirochetes from ticks to mammalian hosts. This work provides a foundation for further elucidation of chemotactic signals sensed by MCP5 that facilitate *B*. *burgdorferi* in evading host defenses.

## Author summary

Lyme disease is the most commonly reported arthropod-borne illness in the US, Europe, and Asia. The causative agent of Lyme disease, *Borrelia or Borreliella burgdorferi*, is maintained in an enzootic cycle involving arthropod vectors (*Ixodes* ticks) and rodent mammalian hosts. Understanding how *B*. *burgdorferi* moves within this natural cycle is crucial for developing new strategies to combat Lyme disease. The complex nature of the enzootic cycle necessitates sensory-guided movement in response to environmental stimuli. *B*. *burgdorferi* possesses a unique and intricate chemotaxis signaling system, with methyl-accepting chemotaxis proteins (MCPs) at its core. MCPs sense environmental signals and guide bacterial movement toward or away from stimuli. This study found that one MCP, MCP5, is highly expressed and differentially regulated during the enzootic cycle by two major regulatory pathways, Hk1-Rrp1 and Rrp2-RpoN-RpoS. MCP5 is essential for mammalian infection and tick transmission by helping spirochetes evade host immune defenses, underscoring its potential as a target for intervention strategies.

## Introduction

Chemotaxis allows motile bacteria to swim towards a favorable environment or away from one that is toxic, which has been well characterized in the two paradigm model organisms *Escherichia coli* and *Salmonella enterica* Typhimurium [1–3]. Bacterial chemotaxis is modulated through a signaling cascade that are composed of chemoreceptors, a coupling protein CheW, a histidine kinase CheA, and a response regulator CheY [3–5]. Bacterial chemoreceptors, also known as methyl-accepting chemotaxis proteins (MCPs), typically contain four functional units, including a periplasmic ligand-binding domain, a transmembrane region, a cytoplasmic HAMP (histidine kinase, adenylyl cyclase, methyl-accepting chemotaxis protein, and phosphatase) domain and a kinase-control module [6,7]. MCPs form trimers of dimers in an array-like structure that typically resides at bacterial cell poles and sense a variety of ligands (e.g., attractants or repellents) [8,9]. Ligand binding to the MCPs, either alone or together with one of the periplasmic binding proteins, promotes a conformational change in the receptor that modulates the activity of CheA. Activated CheA transfers a phosphoryl group to CheY, generating phosphorylated CheY (CheY-P) which in turn interacts with the motor switch complex (also known as C-ring) to control flagellar rotation and locomotion [6]. When the attractant concentration remains stable, bacteria adapt through a process that involves methylation of glutamate residues in the cytoplasmic domains of MCPs [9]. In addition to chemotaxis, MCPs are also implicated in the regulation of biofilm formation [10], flagellum biosynthesis [11], degradation of xenobiotic compounds [12], and production of toxins [13].

*Borrelia* (or *Borreliella*) *burgdorferi*, the causative agent of Lyme disease, is a motile and invasive spirochetal pathogen [14,15]. Motility and chemotaxis are critical for spirochetes to be maintained in the enzootic cycle between tick vectors and vertebrate hosts. When ticks acquire spirochetes from infected vertebrate hosts upon blood feeding, spirochetes are attracted to the tick feeding site by chemotactic signals [16,17]. When infected ticks transmit *B*. *burgdorferi* to naïve vertebrate hosts via feeding, spirochetes replicate, exit the tick gut, move to tick hemocoel, and then migrate to the salivary gland, and are subsequently transmitted to vertebrate hosts [18]. Within vertebrate hosts, *B*. *burgdorferi* cells disseminate from the infection site, and are capable of penetrating host connective tissue and invading various organs such as joints, heart, and nervous system, and causing multi-stage diseases [15,19,20]. In line with its important role in the enzootic cycle, *B*. *burgdorferi* has a unique and complex chemotaxis signaling system [21]. Its genome encodes multiple copies of chemotaxis genes, including two histidine kinases (CheA1 and CheA2), three response regulators (CheY1, CheY2, and CheY3), three coupling proteins (CheW1, CheW2, and CheW3), two sets of chemotaxis adaptation proteins, CheB (CheB1 and CheB2) and CheR (CheR1 andCheR2), and five MCPs (MCP1, MCP2, MCP3, MCP4, and MCP5) and one cytoplasmic chemoreceptor [21–27]. Several motility mutants (e.g., *fliG1*, *flaB*, *fliH*, *fliL*, *flhF*, and *motB*) and chemotaxis mutants (e.g., *cheA1*, *cheA2*, *cheY1*, *cheY2*, *cheY3*,*cheX*, and *cheD*) were reported, and the results demonstrated that motility and chemotaxis are essential for spirochetes to survive and colonize in both ticks and vertebrate hosts [for recent review, see [28]]

How *B*. *burgdorferi* regulates motility and chemotaxis during its enzootic cycle has not been elucidated. In this regard, several regulators and signaling pathways have been identified to coordinately regulate differential gene expression of *B*. *burgdorferi* during the infection [for review, see recent reviews [29,30]]. Among these, Hk1-Rrp1 and Rrp2-RpoN-RpoS pathways play central roles in controlling differential expression of genes critical for tick colonization and mammalian infection, respectively [15,31–33]. The Hk1-Rrp1 two-component signaling pathway senses unknown signals and becomes activated, resulting in the production of a second messenger c-di-GMP [34–36]. This pathway is required for *B*. *burgdorferi* to survive in feeding ticks and complete the enzootic life cycle [34–36]. Hk1-Rrp1 controls the expression of genes important for spirochetal utilization of glycerol, chitobiose, and N-acetylglucosamine, as well as for the process of chemotaxis, motility, and osmolality sensing [34,35,37–42]. On the other hand, the Rrp2-RpoN-RpoS pathway, also called σ^N^-σ^S^ alternative σ factor cascade, is activated by Rrp2 and RpoN (σ^N^) when spirochetes transmit to the mammalian host and during the phase of mammalian infection, resulting in the production of alternative sigma factor RpoS (σ^S^) [43–47]. RpoS, as a global regulator, further activates the transcription of many virulence genes essential for transmission and infectivity in vertebrate hosts, while repressing the expression of genes required for spirochete survival in the tick vector [15,29,43,44].

Compared to other chemotaxis proteins, little is known about the function of MCP chemoreceptors in *B*. *burgdorferi*. Although several chemoattractants of *B*. *burgdorferi* have been identified [16,48–50], the MCP proteins responsible for sensing these attractants remain unknown. The lack of knowledge regarding MCP function has hindered our understanding of how *B*. *burgdorferi* navigates between and within the tick vector and the vertebrate host. In this study, we concentrated on one of the highly expressed MCPs, MCP5, as previous transcriptomic studies showed that mcp5 is differentially expressed and influenced by several regulatory pathways [34,44,51–55]. We demonstrate that its expression is differentially regulated during the enzootic cycle of *B*. *burgdorferi*, controlled by both the Hk1-Rrp1 pathway and the Rrp2-RpoN-RpoS pathway. We further demonstrate that MCP5 plays a pivotal role in mammalian infection by aiding spirochetal evasion of the host’s innate immune response, as well as contributing to spirochetal transmission from ticks to mammals.

## Results

### Structural analyses of *B*. *burgdorferi* MCPs

**Fig 1:**
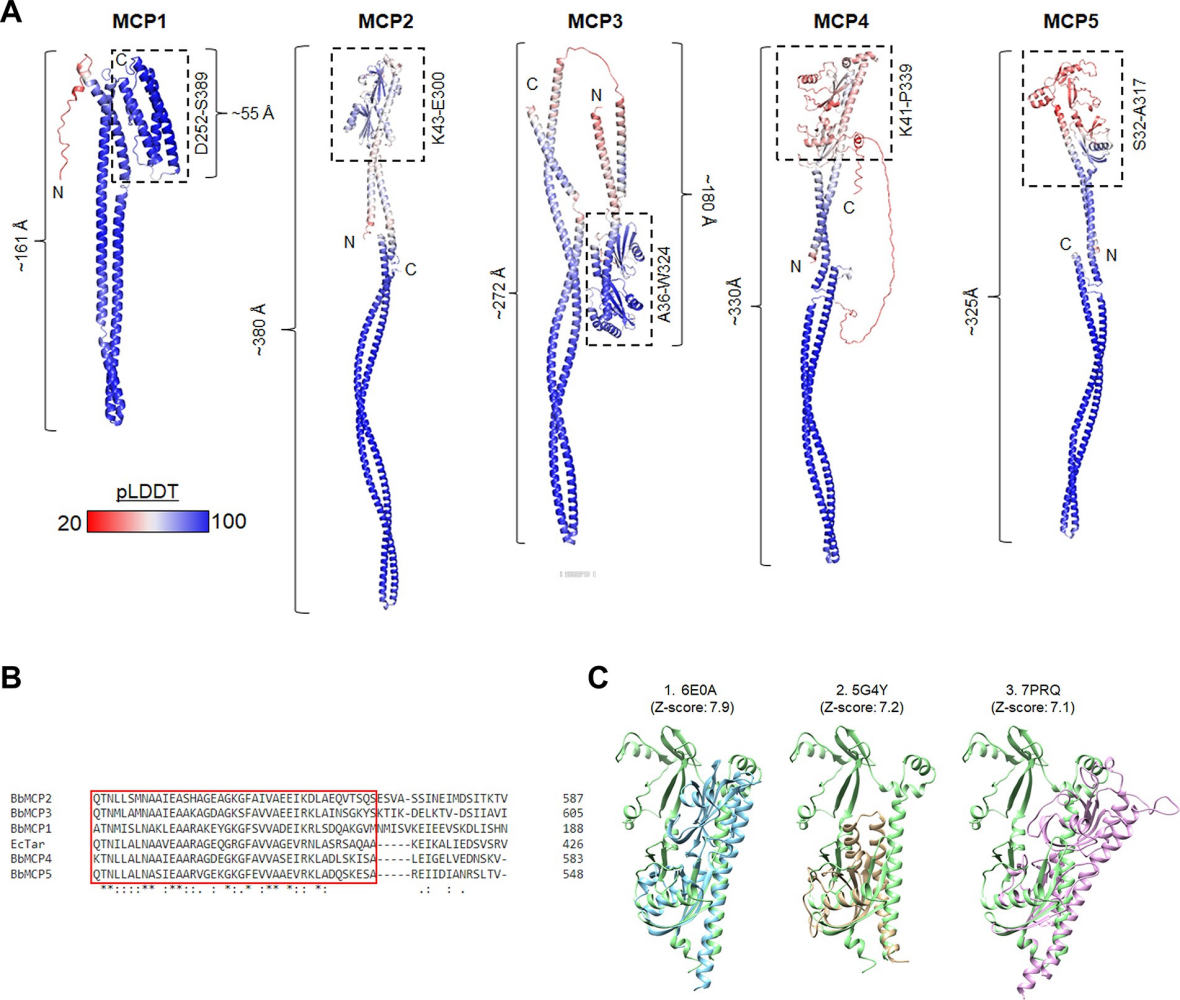
Structural Analysis of MCP1-5 of *B*. *burgdorferi*. **A,** AlphaFold models of MCP1, MCP2, MCP3, MCP4 and MCP5. Protein structures are colored according to their pLDDT scores [60]. For each MCP, the sensor domain is marked with a dotted black box. **B,** Sequence alignment of MCPs 1–5 and EcTar (*E*. *coli* aspartate receptor, Tar). The box in RED is the conserved protein interacting region (PIR) for interacting with the chemosensory arrays. **C**, Structural comparison of the *Bb* MCP5 sensor domain against the top three hits: the ligand-binding domain of *Helicobacter pylori* chemoreceptor TlpA (PDB: 6E0A)[57], the cache-like sensor domain PscD-SD from the plant pathogen *Pseudomonas syringae* (PDB: 5G4Y)[58] and the ligand binding domain of the chemoreceptor PctD from *Pseudomonas aeruginosa* (PDB: 7PRQ)[59]. Structures were identified using the DAHLI server [61].

The genome of *B*. *burgdorferi* encodes five putative chemoreceptors, including MCP1 (BB0578), MCP2 (BB0596), MCP3 (BB0597), MCP4 (BB0680), and MCP5 (BB0681). Our previous study reveals that these MCP proteins form a long, thin array-like structure that resides at the cell poles of *B*. *burgdorferi* [56]; however, their roles in chemotaxis remain largely unknown. To address this question, we first constructed their homology structures using AlphaFold and then compared these structures to their counterparts from other bacteria. Overall, MCPs 2–5 share similar domain composition and structural topology, with MCP1 being the most distant (**Fig 1**). Unlike other MCPs, MCP1 is short (~21.6 nm) and has no N-terminal periplasmic ligand-binding domain. Instead, it has a C-terminal ligand binding domain (**Fig 1A**), suggesting it may function as a cytoplasmic MCP that senses internal signals. MCPs 2–4 form long helical structures with different lengths, ranging from 272 Å to 452 Å (**Fig 1A**). Among these five MCPs, MCP3 is the longest, both in sequence and in receptor length (452 Å, **Fig 1A**). Multiple sequence alignments further revealed that MCPs 1–5 possess a conserved protein-interaction region (PIR) that is typically found in chemoreceptors and is directly interacting with CheA/CheW (also referred as ChaA/CheW binding sites) [7] (**Fig 1B**). Collectively, these results indicate that MCPs 1–5 are canonical chemoreceptors albeit with some sequence and structural variations. In addition, a structure-based similarity search for the *Bb* MCP5 sensor domain resulted in the top three hits in the PDB: the ligand-binding domain of *Helicobacter pylori* chemoreceptor TlpA (PDB: 6E0A) [57], the cache-like sensor domain PscD-SD from the plant pathogen *Pseudomonas syringae* (PDB: 5G4Y) [58] and the ligand binding domain of the chemoreceptor PctD from *Pseudomonas aeruginosa* (PDB: 7PRQ)[59] (**Fig 1C**).

### *mcp5* is highly expressed *in vitro*

**Fig 2:**
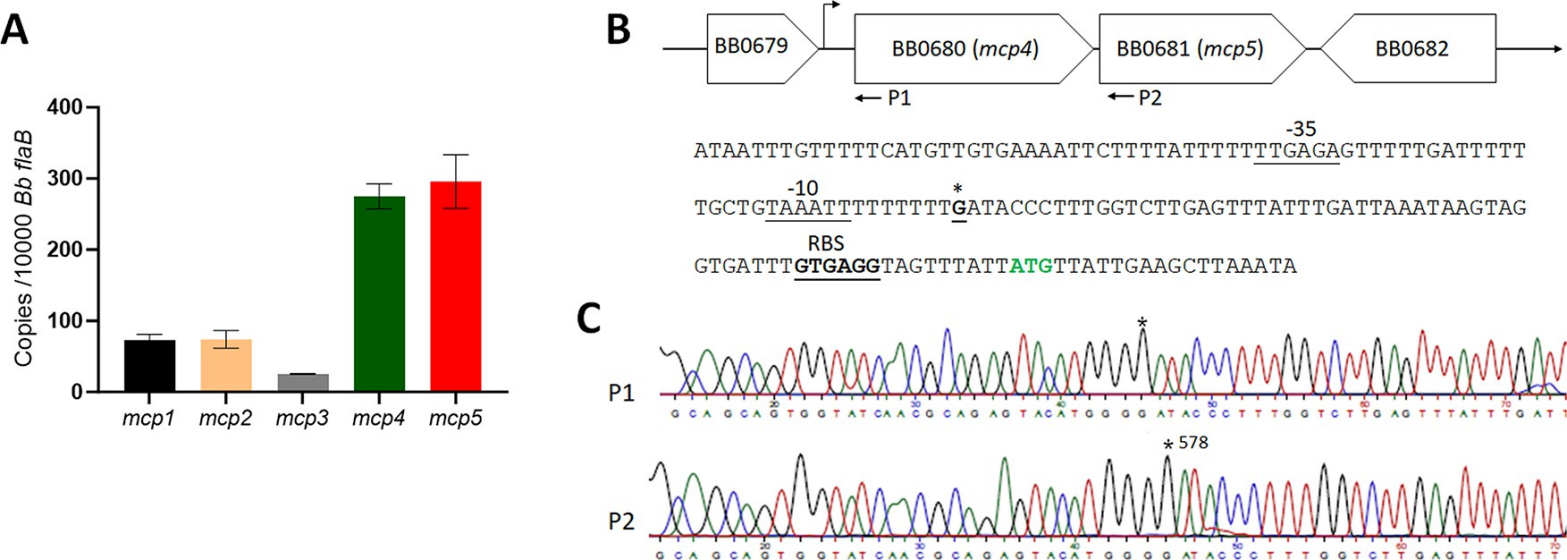
Expression of five *mcp* genes and identification of *mcp5* promoter. (**A**) **qRT-PCR analyses.** Wild-type *B*. *burgdorferi* strain B31M was cultured in the BSK-II medium at 37°C and harvested at the mid-log phase. RNAs were extracted and subjected to qRT-PCR analyses to examine the expressions of all five *mcp* genes. Levels of *mcp* expression relative to the level of *flaB* expression were reported. The bars represent the mean values of three independent experiments. (**B**) Schematic presentation of *mcp4* and *mcp5* in the *B*. *burgdorferi* genome (top) and the promoter sequence at the flanking region of *mcp4* (bottom). The asterisk (*) indicates the transcription start site. The predicted -35 and -10 promoter and RBS sequences are underlined and labeled. ATG in green denotes the start codon of *mcp4*. (**C**) 5′RACE analysis illustrated with a DNA sequencing chromatogram, where the asterisk (*) indicates the +1 position.

To investigate the functions of these MCPs in *B*. *burgdorferi*, we first examined the expression levels of five *mcp* genes in *B*. *burgdorferi* by qRT-PCR. The result showed that *mcp4* and *mcp5* are the two most highly expressed *mcp* genes when spirochetes were cultured under *in vitro* growth conditions (**Fig 2A**). *mcp4* and *mcp5* are adjacent to each other in the *B*. *burgdorferi* genome (**Fig 2B**) [27]. 5′RACE analysis revealed that the transcriptional start sites (TSS) of *mcp4* and *mcp5* are at the same position (G), 62 bp upstream from the ATG start codon of *mcp4* (**Fig 2B and 2C**), indicating these two genes are co-transcribed by the same promoter. A putative -10/-35 σ^70^-like promoter sequence was also identified 6 bp upstream of TSS (**Fig 2B**). Given that *mcp5* is located at the terminus of the *mcp4-mcp5* operon and its genetic inactivation is unlikely to have a polar effect on *mcp4* expression, we focused our investigation on *mcp5* in this study by generating a *mcp5* mutant.

### *mcp5* expression is regulated by environmental cues

**Fig 3:**
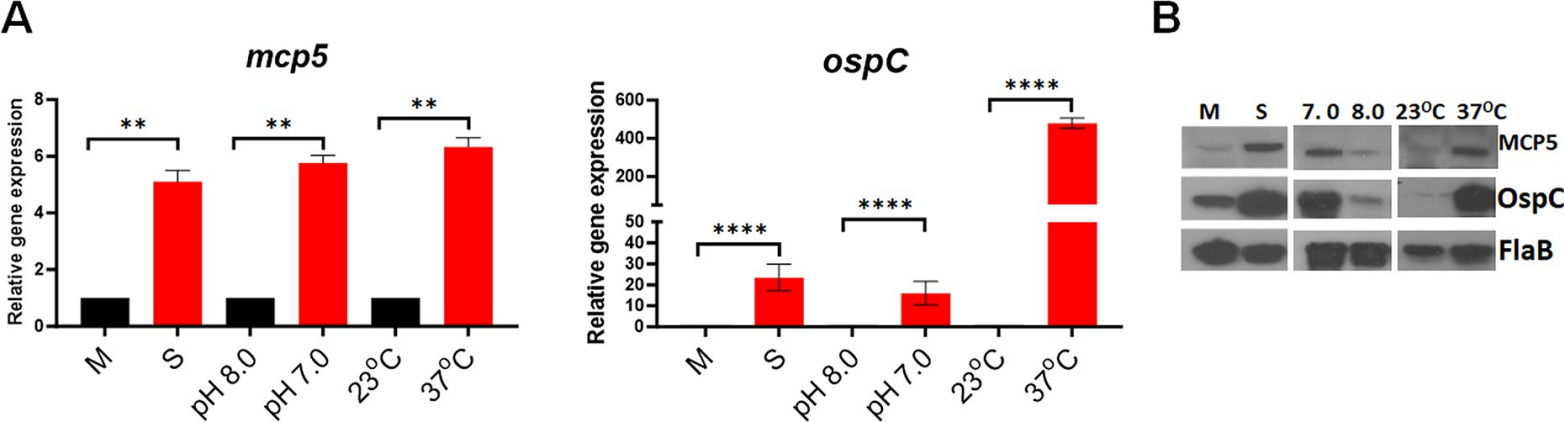
Influence of *mcp5* expression by various environmental cues. (**A**) qRT-PCR analyses. Wild-type *B*. *burgdorferi* strain B31M were cultures in standard BSK-II medium (pH 7.5) at 37°C and harvested at different growth phases [mid-log (M) or stationary (S)], cultured in BSK-II medium under different pH (8.0 vs 7.0), or different temperatures (23°C vs 37°C). RNAs were extracted and subjected to qRT-PCR analyses for the expressions of *mcp5* (left) and *ospC* (right). The relative gene expressions are recorded, with the levels of gene expression in cultures M, pH 8.0, and 23°C normalized to 1.0. The bars represent the mean values of three independent experiments, and the error bars represent the standard deviation. ****, *p* < 0.00001, ** *p* < 0.001 respectively using one-way ANOVA. (**B**) Western blot analyses of the whole cell lysates of spirochetes from (**A**) and (**B**). FlaB was used as a loading control. OspC serves as a control, as it is known to be regulated by temperature, pH and cell density. A representative data from 3 independent experiments is shown here.

Many virulence genes of *B*. *burgdorferi* are differentially expressed in the enzootic cycle and regulated by environmental cues, such as temperature, pH, and cell density[62–64]. Several transcriptomic studies have shown that *mcp4-mcp5* are regulated under various conditions [34,44,51–55]. To investigate whether *mcp5* expression is influenced by culture temperature, pH or cell density, spirochetes were under different culture conditions or harvested at different growth phases, such as mid-log (M) vs stationary (S) growth phases. Harvested spirochetes were subjected to qRT-PCR and immunoblotting analyses. The result showed that the expression of *mcp5* was induced by higher cell density, temperature, and lower pH (37°C, pH 7.0) (**Fig 3A and 3B**), a condition mimicking tick feeding conditions [62–64].

### *mcp5* expression is induced during tick feeding and mammalian infection

**Fig 4:**
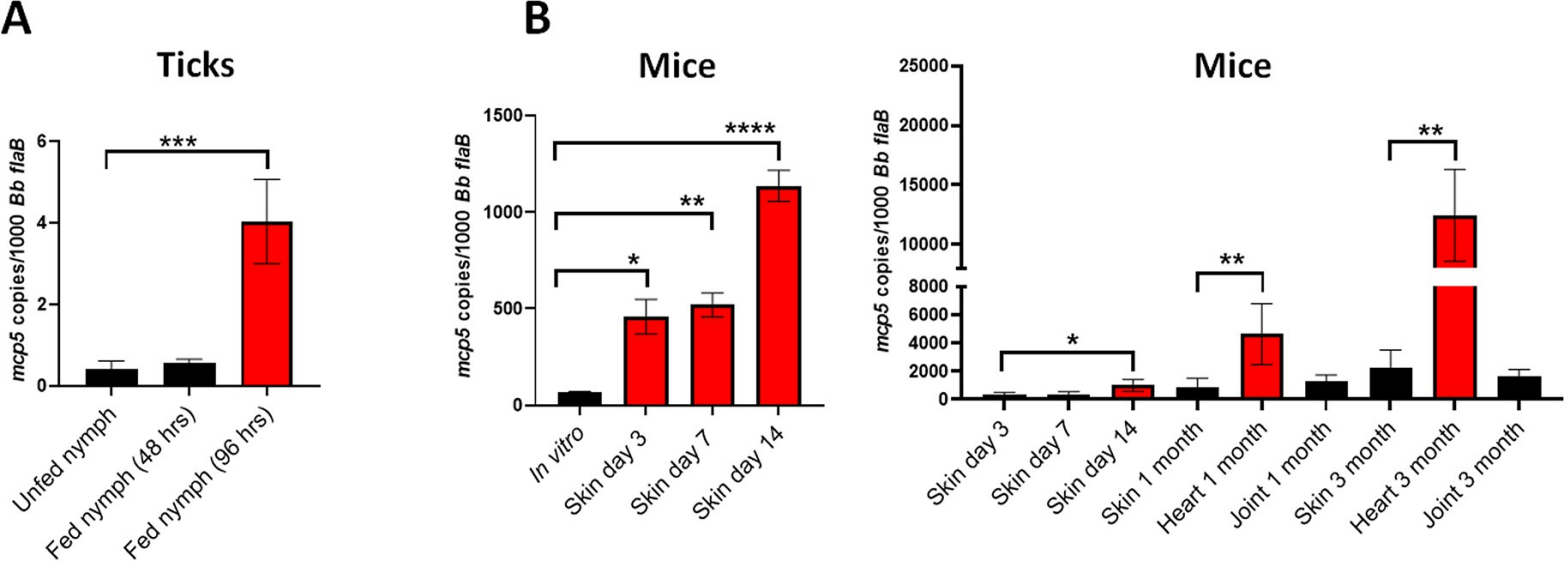
Expression of *mcp5* in the enzootic cycle of *B*. *burgdorferi*. Relative *mcp5* expression in various stages in ticks (**A**) and mice (**B**) was determined by qRT-PCR analyses and reported as copies of *mcp5* per 1,000 copies of the *flaB* transcripts. For gene expression in ticks, infected unfed nymphs or fed nymphs (48- and 96-hours post-feeding) containing *B*. *burgdorferi* were determined by qRT-PCR analyses. The bar represents values from 10 data points, and each data point was generated from 10 unfed nymphs or 1 fed nymph. For mouse experiments, mice were euthanized on days 3, 7, 14, 30 (1 month) and 90 (3 month) post-infection, and skin (site of infection), heart and joint tissues were collected and subjected to RNA extraction followed by qRT-PCR analyses. The bar represents the average values of *mcp5* transcripts calculated from 5 independently infected mice performed in triplicates. The error bars represent the standard deviation. *, *p* < 0.01; **, *p* < 0.001; ***, *p* < 0.0001; ****, *p* < 0.00001 using one-way ANOVA.

To investigate the expression of *mcp5* in the enzootic cycle of *B*. *burgdorferi*, pathogen-free, unfed *I*. *scapularis* larvae were fed on infected mice (C3H/HeN) with wild-type *B*. *burgdorferi* strain B31M via needle inoculation. Fed larvae were allowed to molt to the nymphal stage. Infected flat nymphs were then allowed to feed on naive mice for transmission. Feeding nymphs were collected at 48 or 96 hrs post-feeding. As shown in **Fig 4A**, the *mcp5* transcripts were undetectable in flat nymphs, and blood feeding induced *mcp5* expression at 48 hrs and further induced at 96 hours of feeding. This result indicates that *mcp5* expression is induced upon blood feeding in the transmission phase of the cycle.

To determine the *mcp5* expression the mammalian host, mice were sacrificed at various time points after the infection and skin, joint and heart tissues were collected and subjected to qRT-PCR analyses. The result showed that relative to its expressions in ticks and *in vitro*, the *mcp5* expression had a much higher level of expression in mice (**Fig 4B**). This suggests that *mcp5* expression is further induced when spirochetes replicate in the mammalian host. Interestingly, the *mcp5* expression showed significantly higher levels in heart tissues (1 and 3 months of post infection) than that in other tissues (**Fig 4B**, right panel).

### *mcp5* is regulated by both the Hk1-Rrp1 and Rrp2-RpoN-RpoS pathways

**Fig 5:**
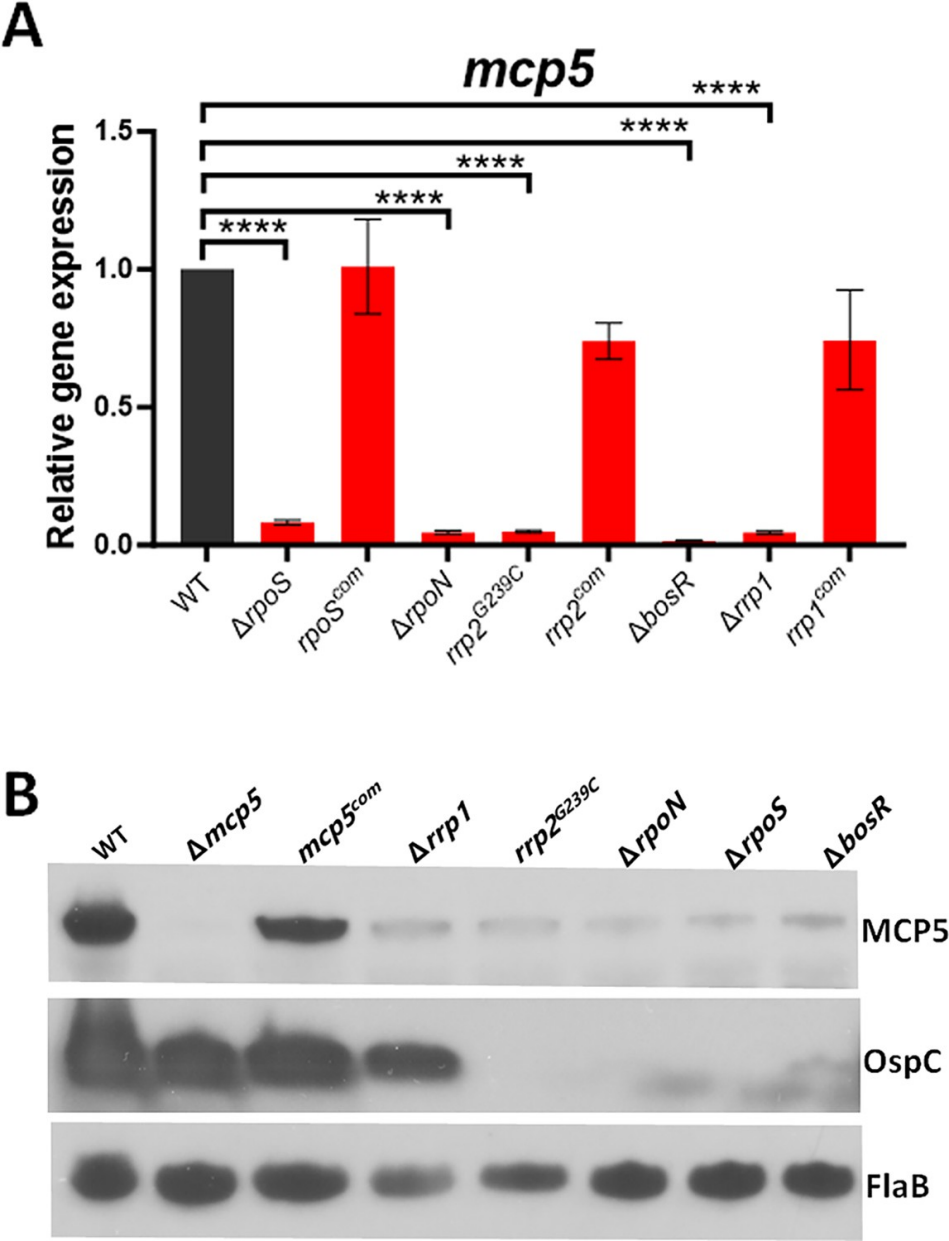
Influences of *mcp5* expression by Rrp1-Hk1 and Rrp2-RpoN-RpoS pathways. **(A)** qRT-PCR analyses. Wild-type *B*. *burgdorferi* strain B31 (WT), *rpoS* mutant (Δ*rpoS*) and its isogenic complemented strain (*rpoS*^*com*^), *rrp2* point mutant (*rrp2*^*G239C*^) and complemented strain (*rrp2*^*com*^), *bosR* mutant (Δ*bosR*), *rrp1* mutant (Δ*rrp1*) and complemented strain (*rrp1*^*com*^) were cultured in BSK-II medium at 37°C and harvested at late-log phase. RNAs were extracted and subjected to qRT-PCR analyses for *mcp5* expressions. The levels of *mcp5* expression in all strains were first normalized with the level of *flaB*. Then, relative levels of *mcp5* expression to the levels in wild-type *B*. *burgdorferi* (which were normalized to 1.0) are reported. The bars represent the mean values of three independent experiments, and the error bars represent the standard deviation. ****, *p* < 0.00001, respectively using one-way ANOVA. (**B**) Immunoblot analyses. Whole cell lysates of various *B*. *burgdorferi* strains were harvested and subjected to immunoblotting using antibodies against MCP5, OspC, or FlaB (loading control). The faint bands detected by anti-OspC in the *rpoS* and *bosR* mutant are likely OspC from an RpoS-independent expression. A representative data from 3 independent experiments is shown here.

Given that the Hk1-Rrp1 pathway and the Rrp2-RpoN-RpoS pathway are the two important pathways that control differential expression of many genes essential for colonization in ticks or infection in mammals [15,31–33], we sought to investigate whether *mcp5* is regulated by these two pathways. To this end, we measured the expression of *mcp5* in various mutants that are defective in these two pathways using qRT-PCR. Our results showed that the *mcp5* expression was significantly downregulated in all the mutants but restored to the wild-type level in their isogenic complemented strains (**Fig 5A**). In consistent with this finding, immunoblot results showed that all the mutants had a diminished level of MCP5 (**Fig 5B**). This finding is consistent with previous transcriptomic studies showing the *mcp5* is regulated by several regulators including Rrp1 and RpoS [34,44,51–55]. These results suggest that the *mcp5* expression is controlled by both the Hk1-Rrp1 and Rrp2-RpoN-RpoS pathways.

### Construction of a *mcp5* mutant and its isogenic complemented strain

**Fig 6:**
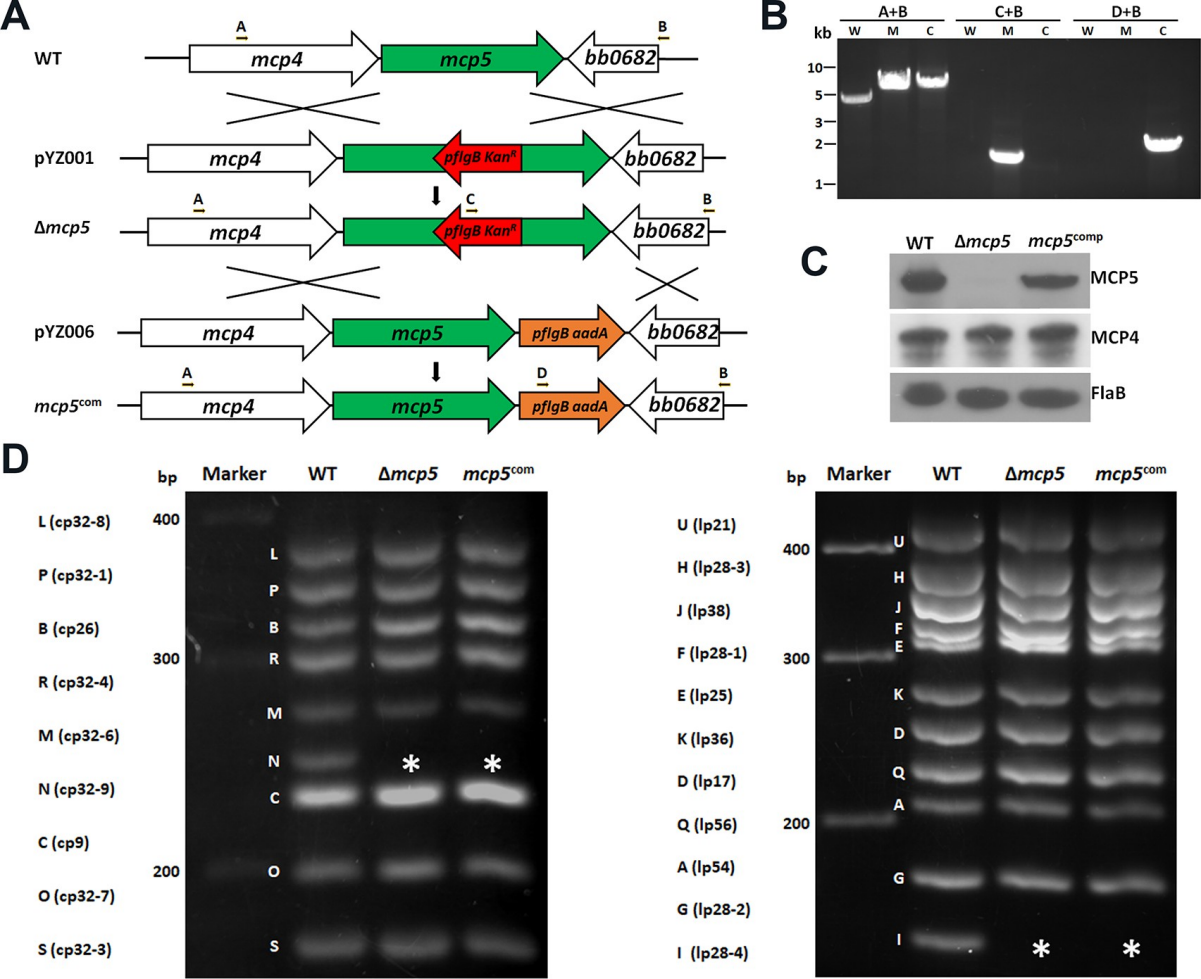
Construction of *mcp5* deletion mutant (Δ*mcp*5) and its isogenic complemented strain (*mcp5*^com^). **(A)** Strategy for constructing the *mcp5* mutant. WT: genomic organization of *mcp5* in *B*. *burgdorferi* genome. pYZ001: the suicide vector used for deletion of *mcp5*. Arrows indicate the primers used for PCR analyses. pYZ006: the suicide vector used for cis-complementing Δ*mcp*5, generating the complemented strain *mcp5*^*com*^. The specific primer pairs used are indicated at the top. **(B)** PCR to confirm inactivation and complementation. W, wild-type *B*. *burgdorferi* strains B31; M, Δ*mcp5*; C, *mcp5*^com^. **(C)** Immunoblotting of MCP4, MCP5 and FlaB levels. *B*. *burgdorferi* strains B31M (WT), Δ*mcp5* and *mcp5*^com^ strains were cultured in BSK-II medium to stationary phase at 37°C. The whole cell lysates were extracted and were probed with antibodies against MCP5, MCP4, and FlaB (loading control). **(D)** Endogenous plasmid profiles. Endogenous plasmid profiles of each strain by multiplex PCR analyses as previously described [66]. cp, circular plasmid; lp, linear plasmid. Letters on the left indicate the bands corresponding to each endogenous plasmid that was defined previously for the *B*. *burgdorferi* strain B31 genome [27, 67]. The asterisk (*) indicates the lost plasmids cp-32-6 and lp28-4 respectively.

To investigate the role of MCP5 in the enzootic cycle of *B*. *burgdorferi*, a *mcp5* mutant and its complemented strain were constructed as illustrated in **Fig 6A**. The loss and restoration of MCP5 production in the *mcp5* mutant (Δ*mcp5*) and complemented strain (*mcp5*^*com*^) were confirmed by PCR (**Fig 6B**) and immunoblotting (**Fig 6C**). Deletion of *mcp5* did not affect the production of MCP4 whose gene was located upstream of *mcp5* (**Fig 6C**), indicating that deletion of *mcp5* has no polar effect on *mcp4* expression. Subsequent endogenous plasmid profile analyses showed that the *mcp5* mutant and its complemented strain lost cp32-6 and lp28-4 (**Fig 6D**). Since these two plasmids are not required for infectivity [65], we proceeded phenotypical characterizations with these two strains. Deletion of *mcp5* had no impact on *B*. *burgdorferi* growth, swimming behaviors, and its response to N-acetylglucosamine (NAG) and rabbit serum, two chemoattractants of *B*. *burgdorferi* (**S1 Fig**) [48,49].

### MCP5 is required for establishing infection in immune competent C3H/HeN mice

**Fig 7:**
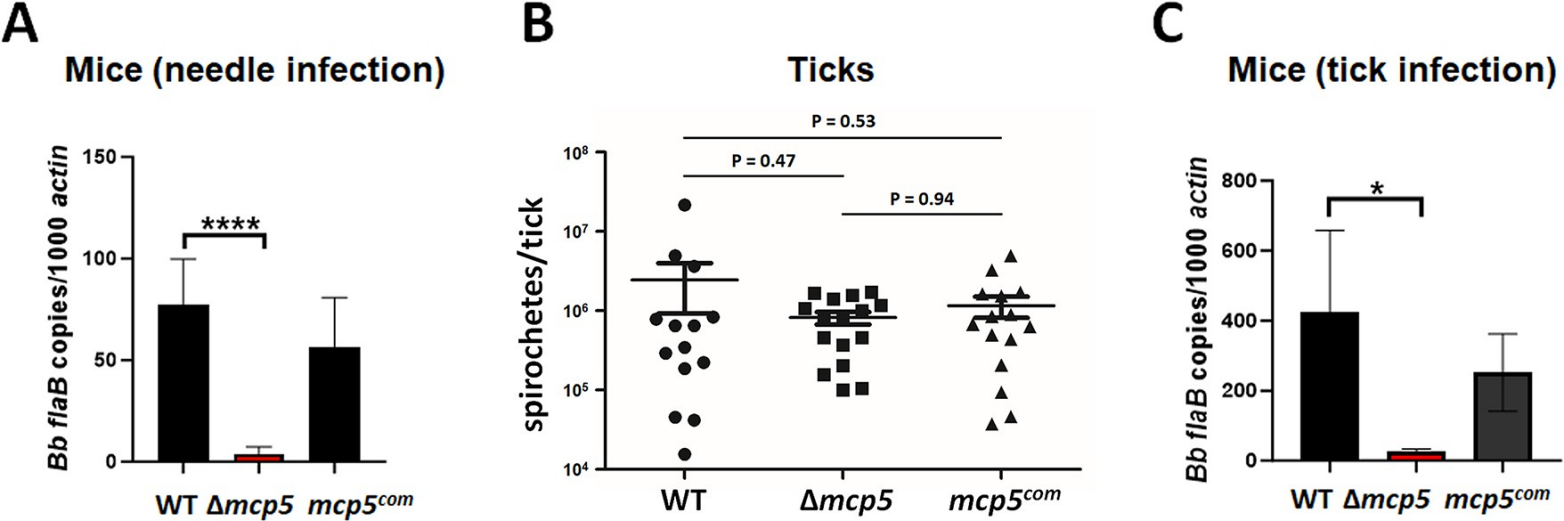
Infection of the *mcp5* mutant in C3H/HeN mice by needle or tick infection. (**A**) Groups of C3H/HeN mice (n = 5) were infected via needle inoculation with 10^5^ cells of WT, Δ*mcp5* and *mcp5*^*com*^. Mice were euthanized 4 weeks post-infection. DNA were extracted from the skin tissues and number of spirochetes were calculated using qPCR. For qPCR analyses, the copy numbers of *flaB* were normalized to those of the mouse actin gene in each DNA sample. The bar represents the mean values of *flaB* DNA copies calculated from 5 mice. (**B**) Unfed nymphs were artificially infected with WT, Δ*mcp5* and *mcp5*^*com*^ by microinjection, and allowed to feed on naïve mice. Fed nymphs collected (n = 15) were lysed and extracted DNA were subjected to qPCR analysis to assess spirochetal loads in ticks. The total copy numbers of *flaB* were reported as per tick DNA sample. The bar represents the mean values of *flaB* DNA copies calculated from 15 fed nymphs. (**C**) Groups of C3H/HeN mice (n = 5) infected by tick bites were euthanized upon tick repletion and skin tissues from the site of tick bite were subjected to qPCR analyses as described in (**A**). *, *P* < 0.01; ****, *P* < 0.00001 using one-way ANOVA.

To examine the potential role of MCP5 in the infectious cycle of *B*. *burgdorferi*, groups of C3H/HeN mice were needle inoculated with WT, Δ*mcp5* and *mcp5*^*com*^ strains with a dose of 1 X 10^5^ spirochetes/mouse. Ear punch biopsies were collected at 2-, 3-, and 4-weeks post-infection and cultured in BSK-II medium for the presence of spirochetes. At 4-weeks post-infection, all mice were sacrificed and several mouse tissues including ear, joint, heart, skin, and bladder were collected and cultured. Virtually all cultures of tissues from mice inoculated with the wild-type or the complemented strains were positive for *B*. *burgdorferi* growth. In contrast, only 1 out of 50 cultured mouse tissues showed culture positive from mice infected with the *mcp5* mutant (**Table 1**). To substantiate these observations, spirochetal loads in skin tissues were determined by qPCR. The result showed that the tissues from mice inoculated with the *mcp5* mutant had virtually no detectable or low numbers of spirochetes (**Fig 7A)**. These data indicate that MCP5 is a virulence factor required for *B*. *burgdorferi* to establish mammalian infection.

**Table 1:** Infection of the *B*. *burgdorferi mcp5* mutant in immunocompetent mice (C3H/HeN) (4 weeks of post-infection).

Strain	Dose	Heart	Joint	Ear	Bladder	Spleen	No. of culture positive/No. of tissues*
B31M	1x10^5^	10/10	10/10	10/10	10/10	10/10	50/50^£^
Δ*mcp5*	1x10^5^	1/10	0/10	0/10	0/10	0/10	1/50^£^
*mcp5* ^*com*^	1x10^5^	7/10	9/10	9/10	6/10	10/10	41/50

*Tissues harvested at 4 weeks of post-infection

^£^ *p* < 0.001 using Fisher’s exact two-tail test

To determine the role of MCP5 in ticks, flat nymphs were artificially infected with wild-type *B*. *burgdorferi*, the *mcp5* mutant and complemented strains via microinjection [68]. Ticks were then allowed to feed on naïve C3H/HeN mice. Engorged nymphs were collected, and spirochetal loads were assessed by qPCR. The results showed that no significant difference was observed in the estimated spirochetal numbers among ticks harboring each strain (**Fig 7B)**, suggesting that the *mcp5* mutant is capable of replicating in tick guts during blood meal. To assess the efficiency of transmission from ticks to mice, mouse skin tissues at the site of tick bites immediately upon tick repletion were harvested and subjected to qPCR analyses. The result showed that virtually no or low amounts of *B*. *burgdorferi* DNA were detected in mouse skin tissues from mice infected with ticks carrying the *mcp5* mutant (**Fig 7C**), suggesting that although MCP5 is dispensable for replication in ticks, it is required for spirochetes to transmit to the mammalian host.

**Fig 8:**
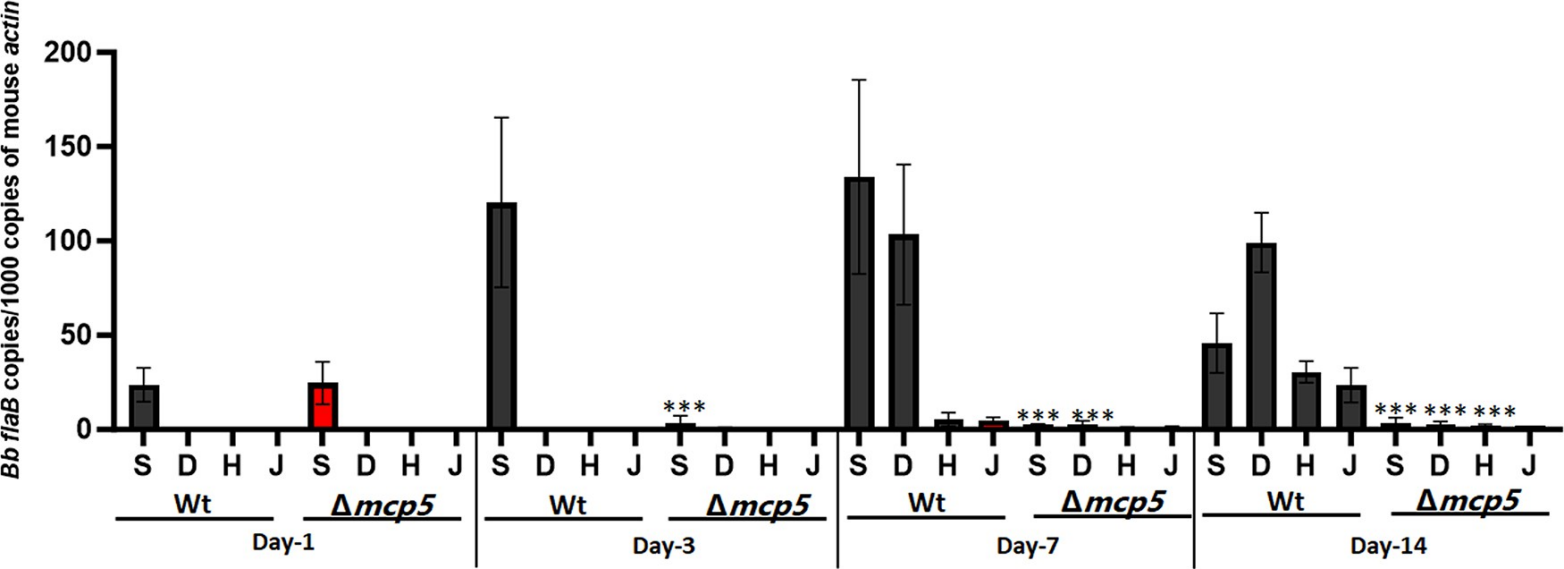
Relative number of *B*. *burgdorferi* genomes in mouse tissues as determined by qPCR. Groups of C3H/HeN mice (n = 4 per clone per time point) were needle infected with 1×10^5^ WT and Δ*mcp5*, and euthanized after day 1, 3, 7 and 14 days of post-infection. Mouse tissues including skin from the site of infection (**S**), skin at the distant site (**D**), heart (**H**) and joint (**J**) were processed for qPCR analyses. The calculated number of *flaB* copies was normalized to that of mouse actin. The bar represents the mean values of *flaB* DNA copies calculated from each of 4 mouse tissues. ***, *P* < 0.0001; using one-way ANOVA.

The inability to establish infection in mice by the *mcp5* mutant could be due to a defect in early colonization or in dissemination. To further investigate the nature of contribution of MCP5 to mammalian infection, immune competent C3H/HeN mice inoculated with wild-type and the *mcp5* mutant were examined at various days post-infection (i.e., day 1, 3, 7 and day 14). At day 1 post-infection, the spirochetal numbers of all strains were similar in the skin tissues of inoculation site (**Fig 8**). On day 3 post-infection, wild-type spirochetes showed increased numbers at the site of infection and were detected in distal mouse tissues at day 7 and day 14 post-infection. In contrast, at day 3 post-infection, the numbers of *mcp5* mutant at the site of inoculation were significantly reduced (50-fold less than those of the wild-type strain); no mutant spirochetes were detected in distal mouse tissues at day 7 and day 14 post-infection, suggesting that the *mcp5* mutant spirochetes were quickly cleared at the site of inoculation as early as day 3 post-infection.

### The *mcp5* mutant disseminated and established infection in NOD scid gamma (NSG) mice

**Fig 9:**
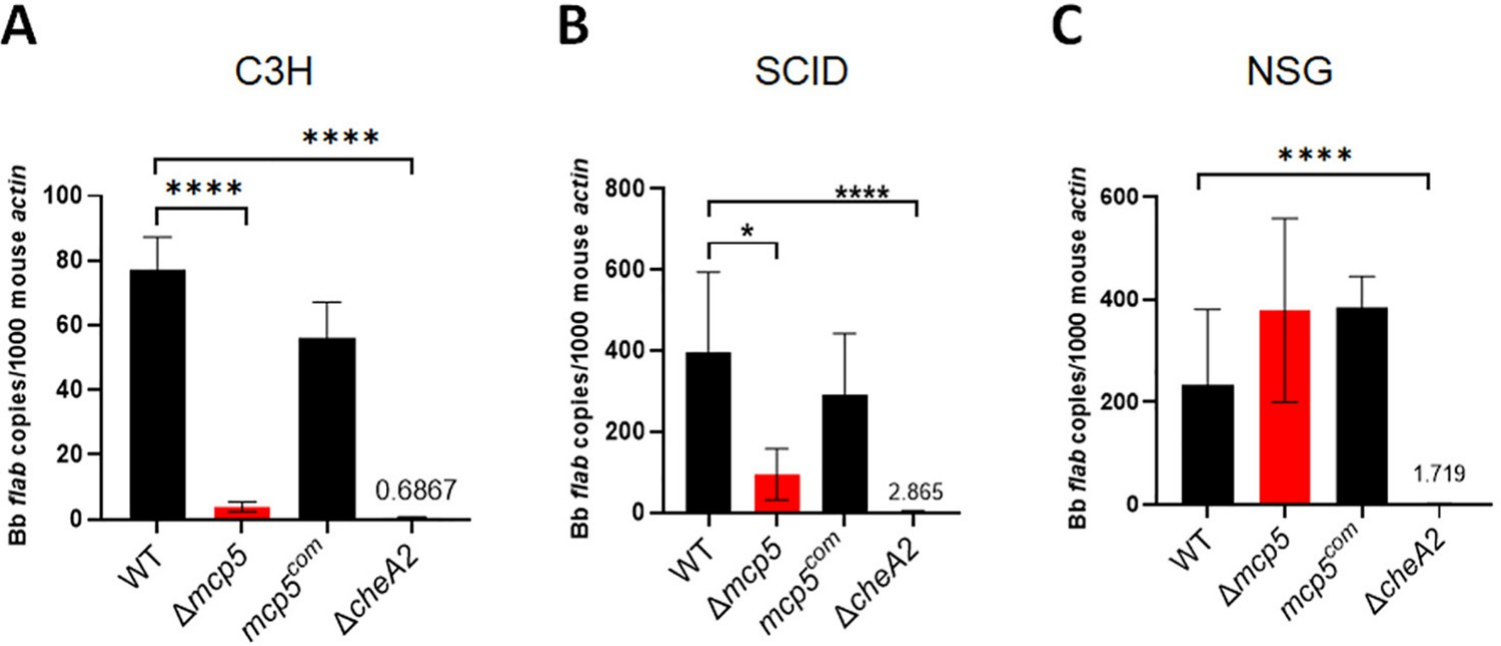
Spirochetal loads in infected SCID and NSG mice. Groups of C3H mice (n = 5 for each data point) (**A**) SCID mice (n = 5 for each data point) (**B**) and NSG mice (n = 5 for each data point) (**C**) were inoculated with 10^5^ of WT, Δ*mcp5*, *mcp5*^*com*^, and Δ*cheA2*, strains. Mice were euthanized at 4 weeks post infection and skin (site of infection) were collected and then were subjected to DNA extraction followed by qPCR analyses. The bar represents the mean values of *flaB* DNA copies calculated from 5 infected mice performed in triplicates. *, *p* < 0.01, ****, *p* < 0.00001; using one-way ANOVA.

To gain further insight into the role of MCP5 in mammalian infection, we assessed the infectivity of the *mcp5* mutant in Severe Combined immunodeficient SCID mice that lack adaptive immunity (deficient in B and T cells) [69]. Similar with observations in C3H/HeN mice, the *mcp5* mutant exhibited significantly lower spirochetal loads at the inoculation site in SCID mice (**Fig 9A** and **9B**), indicating that innate immunity alone may be sufficient to clear the *mcp5* mutant. We then further tested the infectivity of the *mcp5* mutant in NSG mice (NOD-*scid* IL2Rgamma^null^), which are severely immunodeficient due to both *scid* mutation and a null mutation in the IL2 receptor common gamma chain (IL2Rgamma^null^) [70]. The absence of IL2Rγ blocks natural killer (NK) cell differentiation [71]. Thus, in addition to adaptive immunity defects, NSG mice also lack NK cells [70]. The result revealed that the *mcp5* mutant displayed spirochetal burdens similar to that of wild-type *B*. *burgdorferi* at the inoculation site (**Fig 9C**) and was able to disseminate to all tissues examined (**Table 2**), suggesting a crucial role in innate immunity in clearing the *mcp5* mutant.

**Table 2:** Infectivity of the *mcp5* mutant in wild-type and NK cell depleted mice.

	*Borrelia* strain	Dose	Site of inoculation	Heart	Joint	Ear	No. of tissues positive/Total No. of tissues
**C3H mice** *	Δ*mcp5*	1x10^5^	2/14				2/14^€^
	Δ*cheA2*	1x10^5^	0/3				0/3
**NK cell-depleted C3H mice** *	Δ*mcp5*	1x10^5^	4/4				4/4^€^
**WT SCID mice** *	B31M	1x10^5^	9/9	9/9	9/9	9/9	36/36
	Δ*mcp5*	1x10^5^	3/11	1/11	0/11	0/11	4/44^£^
	*mcp5* ^*com*^	1x10^5^	7/9	9/9	9/9	8/9	33/36
	Δ*cheA2*	1x10^5^	0/3	0/3	0/3	0/3	0/3
**NK cell-depleted SCID mice** *	B31M	1x10^5^	3/3	3/3	3/3	3/3	12/12
	Δ*mcp5*	1x10^5^	3/3	2/3	2/3	3/3	10/12^£^
	*mcp5* ^*com*^	1x10^5^	3/3	3/3	3/3	3/3	3/3

*For C3H mice, tissues were harvested at Day 8 post-infection; for SCID mice, tissues harvested at Day 18 post-infection.

^€^ *p* = 0.05

^£^ *p* < 0.001 using Fisher’s exact two-tail test

Given that MCP5 is a predicted chemoreceptor, we compared the *mcp5* mutant with the *cheA2* mutant, which lacks CheA2 histidine kinase essential for the chemotaxis of *B*. *burgdorferi*. Previous studies have shown that the *cheA2* mutant is non-chemotactic to attractants and incapable of infecting C3H and SCID mice [72]. The results showed that, unlike the *mcp5* mutant, the *cheA2* mutant was unable to establish infection or disseminate in NSG mice (**Fig 9C** and **Table 2**). These findings suggest that the mcp5 mutant retains its chemotactic function in NSG mice and that MCP5-associated chemotaxis may play a role in evading the innate immune responses present in C3H and SCID mice.

### NK cell depletion restores the infectivity of the *mcp5* mutant in SCID mice

**Fig 10:**
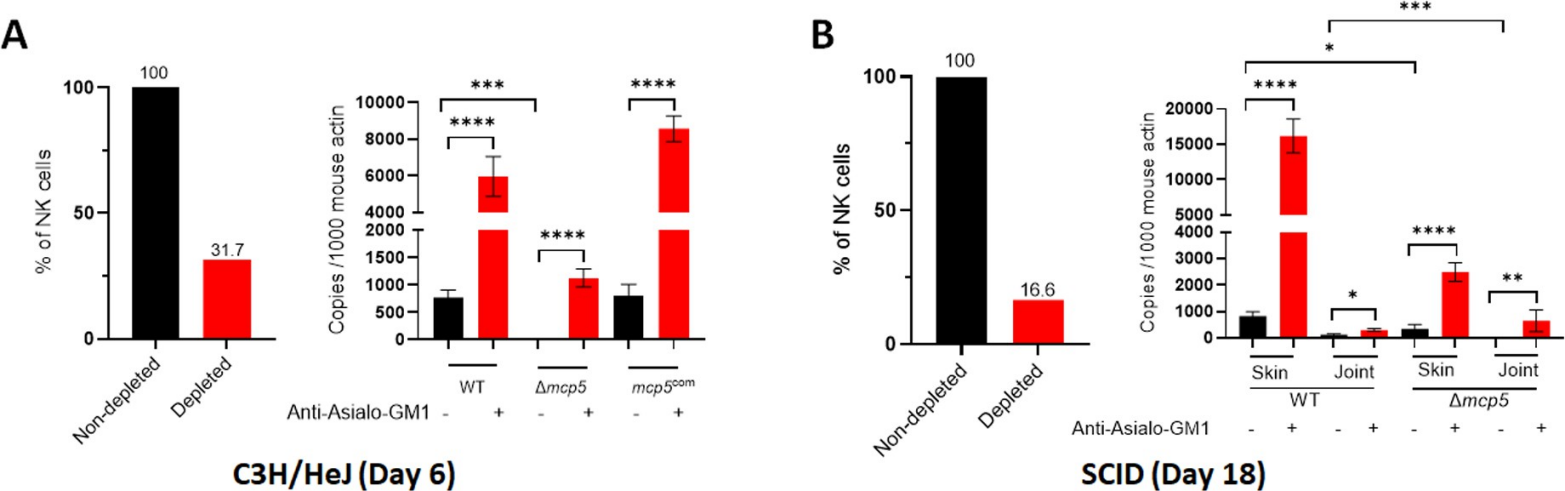
NK cell depletion and spirochetal burden in C3H and SCID mice. Groups of C3H/HeN (**A**) and SCID (**B**) mice were intraperitoneally injected with the Anti-Asialo GM1 antibody to deplete NK cell. Single cellw from the spleen tissues were prepared and stained with anti-CD45, -CD3, and -CD49b antibodies. Percentages of NK cell (CD45+CD3-CD49b+) were assessed using flow cytometric analysis and percentages of reduction in NK cells were determined (**Left panels**). NK cell depleted and non-depleted mice (n = 3) were inoculated with 10^5^ of WT *B*. *burgdorferi*, Δ*mcp5*, and *mcp5*^com^ strains. Mice were euthanized at 6-day post infection for C3H/HeN mice, or at 18-day post-infection for SCID mice. Skin tissues at the inoculation site (for both C3H/HeN and SCID mice) and joints (for SCID mice) were collected and then were subjected to DNA extraction followed by qPCR analyses (**Right panels**). The bar represents the mean values of *flaB* DNA copies calculated from 3 infected mice performed in triplicates. Black bar represents non-depleted mice; Red bar represents NK cell-depleted mice. *, *p* < 0.01;.**, *p* < 0.001; ***, *p* < 0.0001; ****, *p* < 0.00001; using one-way ANOVA.

The above data indicate that the *mcp5* mutant is unable to establish infection in C3H and SCID mice but can infect NSG mice. Given that the primary immunological difference between SCID and NSG mice is the absence of NK cells in NSG mice, we hypothesize that NK cells in C3H and SCID mice are crucial for clearing the *mcp5* mutant. To test this, we depleted NK cells in C3H and SCID mice by administering anti-Asialo Ganglioside-GM1 (ASGM1) antibodies, which bind the ASGM1 glycolipid on NK cell surfaces, effectively depleting NK cells *in vivo*. The efficacy of NK cell depletion was confirmed by flow cytometry, measuring the percentage of CD49b+ NK cells, a surface marker specific for NK cells (**S2 and S3 Figs)**. Treatment with the NK cell-depleting antibody reduced CD49b+ NK cells by 69% in C3H mice and by 84% in SCID mice (**Fig 10A** and **10B, left**).

Following NK cell depletion, mice were challenged with wild-type *B*. *burgdorferi*, the *mcp5* mutant, or the complemented strain. C3H mice were sacrificed six days post-inoculation to assess acute infection, and SCID mice were sacrificed 18 days post-inoculation to assess disseminated infection. Spirochetal burdens at the inoculation site (skin) and in joints were quantified by qPCR. Results showed that in C3H mice, NK cell depletion significantly increased bacterial loads of wild-type *B*. *burgdorferi* at the inoculation site (**Fig 10A, right**). More importantly, it also increased the level of the *mcp5* mutant to that of wild-type *B*. *burgdorferi* in non-depleted C3H mice. All four NK cell-depleted C3H mice showed culture-positive of the *mcp5* mutant (**Table 2**). Similarly, NK cell-depleted SCID mice displayed dramatically increased bacterial loads of both the wild-type strain and the *mcp5* mutant at the inoculation site (**Fig 10B, right**). Increased bacterial loads were also observed in joint tissues of NK cell-depleted SCID mice, although the increase was less pronounced than in skin tissues. Notably, NK cell depletion led to disseminated infection in SCID mice by the *mcp5* mutant, as shown by culture positivity in skin, heart, joint, and ear tissues (**Table 2**). These findings further support the critical role of NK cells in clearing the *mcp5* mutant.

### The *mcp5* mutant infection induced an enhanced production of gamma interferon by NK cells

**Fig 11:**
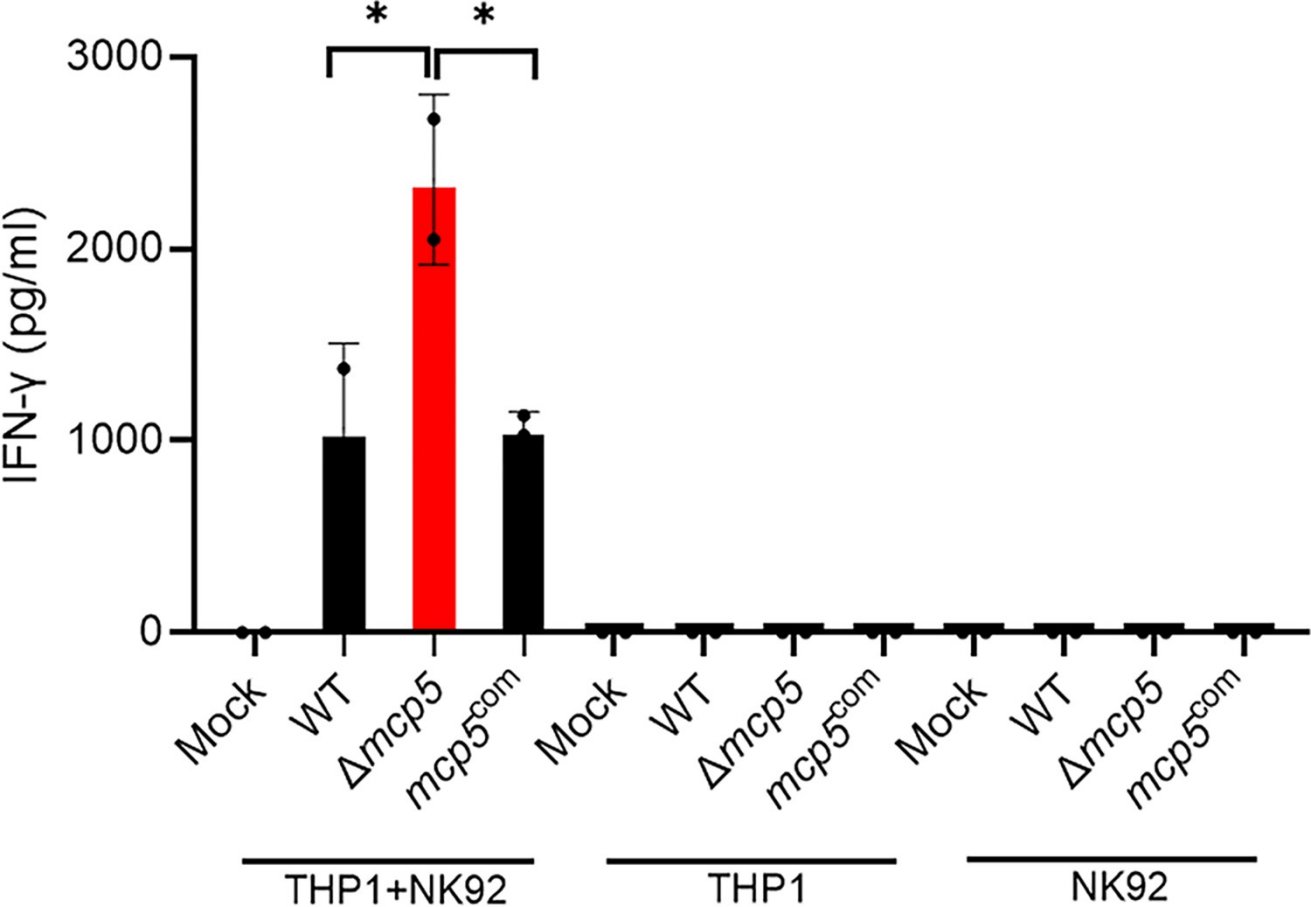
Enhanced IFN-γ production of NK cells by the *mcp5* mutant. THP-1 cells were differentiated into macrophages by treatment with PMA (5ng/ml for 24 hours). The differentiated macrophages (1*10^5 cells/well) were then infected with WT *B*. *burgdorferi*, Δ*mcp5*, or *mcp5*^com^ at a multiplicity of infection (MOI) of 10 for 6 hours. After infection, the cells were washed and co-cultured with NK-92 cells at a 1:1 ratio (1*10^5 cells/well) for 24 hours. The THP-1 and NK-92 cells cultured alone, with and without infection with *B*. *burgdorferi* were used as controls. The concentration of IFN-γ in the culture supernatant was quantified using ELISA. The bars represent the mean IFN-γ levels from two independent experiments, each performed in triplicate. *, *p* < 0.01; using one-way ANOVA.

To understand how NK cell depletion enables the *mcp5* mutant to establish infection in mice, we examined whether MCP5 influences NK cell function in response to *B*. *burgdorferi* infection. NK cells are key components of the innate immune system, defending against infections either by directly killing pathogen-infected cells or by recruiting phagocytic cells through the production of gamma interferon (IFN-γ) [73, 74]. NK cell activation can be triggered by bacterial pathogen-associated molecular patterns (PAMPs), which are detected by accessory cells like dendritic cells (DCs), macrophages, and monocytes [75]. To assess NK cell activation, we conducted co-culture experiments using human NK cells (NK-92) and macrophages (THP-1) exposed to *B*. *burgdorferi* infection and measured IFN-γ levels in the supernatant as an indicator of NK cell activation. The results showed that neither NK cells nor macrophages alone produced detectable levels of IFN-γ when incubated with *B*. *burgdorferi* (**Fig 11**). However, when NK cells were co-cultured with macrophages infected with wild-type *B*. *burgdorferi*, IFN-γ production was readily detected. Notably, IFN-γ levels were significantly higher when NK cells were co-cultured with macrophages infected with the *mcp5* mutant. These findings suggest that infection with the *mcp5* mutant leads to enhanced IFN-γ production by NK cells.

**Fig 12:**
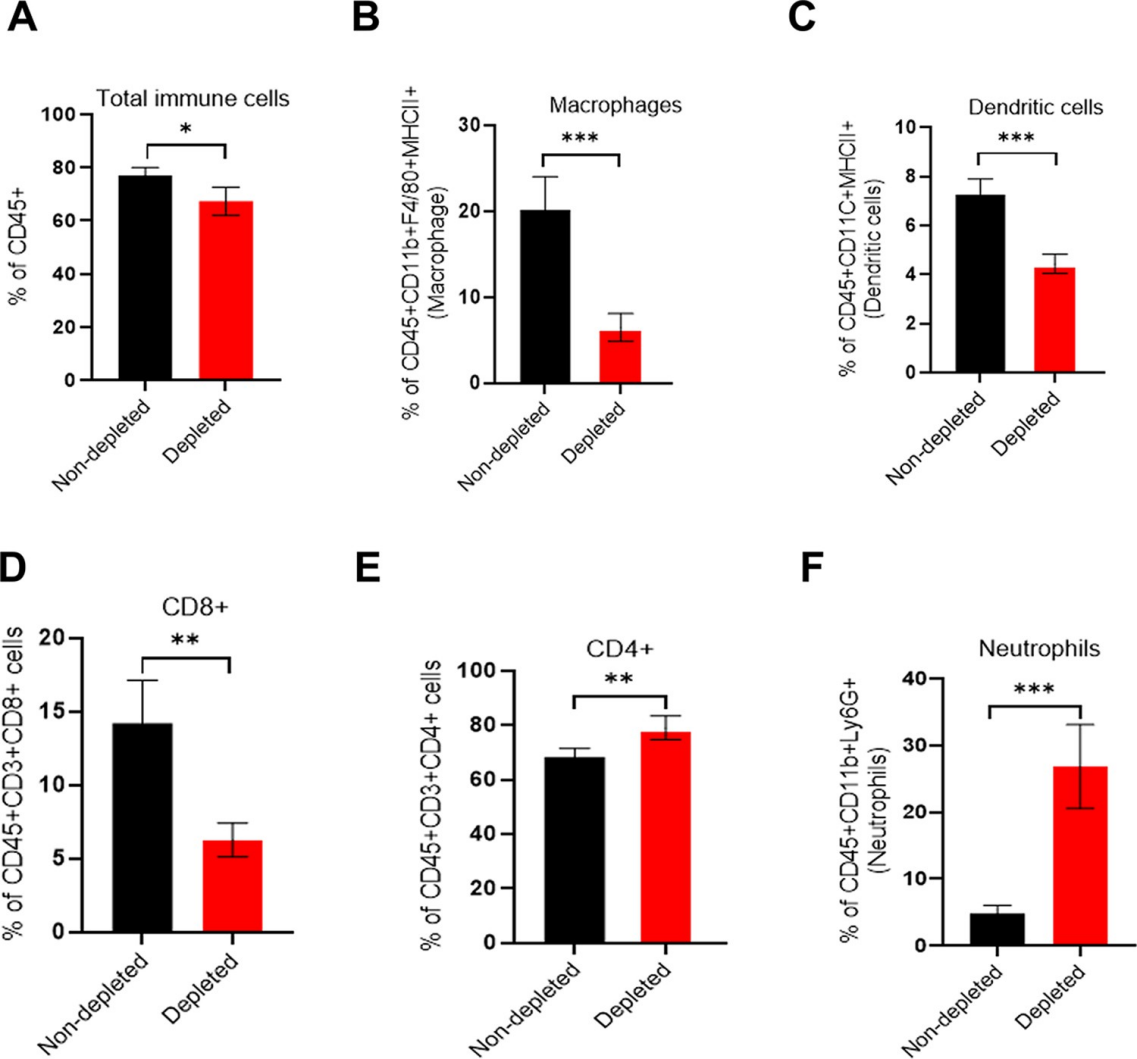
Immune cell infiltration at the site of inoculation. Groups of C3H mice with either NK cell-depleted or non-depleted (n = 4) were infected with Δ*mcp5* (1*10^5^/mouse). Mice were euthanized at 8 days post-infection and the skin tissues at the site of infection were harvested. Single cells were prepared from the skin samples and stained with anti-**CD45, -CD3, -CD4, -CD8a, -CD11b, -F4/80, -Ly6C, -Ly6G, -CD11c,** and **-**I-A/I-E Abs. The percentage of total white blood cells (CD45+) (**A**), macrophages (CD45+CD11b+F4/80+I-A/I-E+) (**B**), dendritic cells (CD45+CD11C+I-A/I-E+) (**C**), CD8+ T cells (CD45+ CD3+CD8+) (**D**), CD4+ T cells (CD45+ CD3+CD4+) (**E**), and neutrophils (CD45+ CD11b+Ly6G+) (**F**), were determined using Flow-cytometry analysis. The bar represents the mean values of th*e* percentage of immune cells calculated from 4 infected mice. *, *p* < 0.01;.**, *p* < 0.001; ***, *p* < 0.0001; ****, *p* < 0.00001; students t-test.

To understand how NK cell depletion enables the *mcp5* mutant to establish infection at the inoculation site in C3H mice, we conducted flow cytometry assays to assess immune cell recruitment in NK cell-depleted and non-depleted C3H mice infected with the *mcp5* mutant. Results showed a significant reduction in macrophages, dendritic cells, and CD8+ T cells in NK cell-depleted mice. Surprisingly, an increase in neutrophils was observed (**Fig 12**). No changes were observed in CD4+ T cell levels. These findings suggest that activated NK cells are crucial for recruiting macrophages, dendritic cells, and CD8+ T cells and these immune cells play an important role in clearing the *mcp5* mutant.

## Discussion

The complex nature of the enzootic cycle of *B*. *burgdorferi* necessitates sensory-guided movement in response to changes in stimuli. Approximately 6% of the genes encoded in the *B*. *burgdorferi* genome contributes to motility and chemotaxis, underscoring their importance in spirochetal complex life cycle [27]. Although much is known about the motility and chemotaxis of *B*. *burgdorferi*, the knowledge about the chemoreceptors MCPs has been lacking [21, 28]. In this study, we provide evidence that *mcp5* is one of the most abundant and differentially expressed *mcp* genes during the enzootic cycle of *B*. *burgdorferi*. We further demonstrate that MCP5 is indispensable for *B*. *burgdorferi* to establish infection in vertebrate hosts, likely by playing an important role in evading host innate immunity.

Previous transcriptomic studies have shown that *mcp4-mcp5* are differentially expressed under various conditions and controlled by various regulators including the Hk1-Rrp1 pathway and the Rrp2-RpoN-RpoS pathway [34,44,51–55]. During the enzootic cycle of *B*. *burgdorferi*, the Rrp2-RpoN-RpoS pathway is activated during the transmission of spirochetes from ticks to vertebrate hosts and during the infection in vertebrate hosts. This pathway functions as a “gatekeeper” during tick feeding, turning on genes required for spirochetes to establish infection in vertebrate hosts. The inability of the *rrp2*, *rpoN*, *rpoS*, and *bosR* mutants defective in the Rrp2-RpoN-RpoS pathway to express *mcp5* clearly indicates that *mcp5* expression is controlled by Rrp2-RpoN-RpoS during *in vitro* growth conditions (**Fig 5**). Several lines of evidence suggest that *mcp5* expression is also controlled by Rrp2-RpoN-RpoS during the enzootic cycle of *B*. *burgdorferi*. Firstly, *mcp5* is differentially expressed during transmission and mammalian infection, correlating with the activation of the Rrp2-RpoN-RpoS pathway. Secondly, MCP5 is required for mammalian infection and for transmission from ticks to mice. How does RpoS control mcp5 expression? Promoter mapping revealed that *mcp5* is transcribed from a σ^70^-type promoter located upstream of *mcp4*, suggesting that *mcp5* is directly regulated by a yet-to-be-identified regulator within the RpoS regulon, or directly by RpoS, given that the promoter sequences between σ^S^ and σ^70^ are nearly indistinguishable.

The finding that expression of *mcp5* is dependent on both the Hk1-Rrp1 pathway and the Rrp2-RpoN-RpoS pathway, as Hk1-Rrp1 is activated in spirochetes replicating in feeding ticks during acquisition and in spirochetes colonizing unfed ticks [34–36]. Interestingly, previous global transcriptomic analyses have also shown that *mcp5* is one of the genes whose expression is affected by both Hk1-Rrp1 and Rrp2-RpoN-RpoS [34,38,46,52,76,77]. Given that we and others previously reported that there is an interplay between Hk1-Rrp1 and Rrp2-RpoN-RpoS [51,78,79], i.e., Rrp1 can regulate *rpoS* expression, it remains to be determined whether Hk1-Rrp1 regulates *mcp4-mcp5* expression via RpoS, or via an RpoS-independent mechanism. In addition, MCP5 appears to be dispensable for replication in the nymphal gut during transmission but is important for spirochetal migration to mice (**Fig 7**). Whether this is a defect in transmigration from the tick midgut, survival in tick hemolymph, migration to the tick salivary glands, or deposition into mouse dermis, remains to be determined. Thus, it will also be interesting to examine whether MCP5 plays a role in acquisition by ticks and colonization within ticks. Several motility and chemotaxis mutants have been reported to have various phenotypes in ticks. Like the *mcp5* mutant, the *cheY2* mutant can survive in nymphs but fails to transmit *B*. *burgdorferi* from ticks to mice [80]. A similar phenotype was recently found in the *cheA1* mutant [81]. On the other hand, the *motB* or *cheY3* mutant has reduced spirochetal numbers in feeding ticks [82,83]. It was speculated that the *motB* or *cheY3* mutant spirochetes could not achieve certain interactions that allows protection against bactericidal factors present in the ingested blood meal in the tick midgut [21].

Our infection study revealed that the *mcp5* mutant was unable to establish infection or disseminate in either immune-competent or SCID mice but was detectable in all tested tissues in NSG mice (**Tables 1 and 2**). SCID mice have a defect in double-strand DNA repair that prevents T cell receptor (TCR) and B cell receptor (BCR) recombination, resulting in a lack of mature T and B cells. However, they retain components of the innate immune system, including natural killer (NK) cells, macrophages, granulocytes, and complement proteins [69]. The inability of the *mcp5* mutant to infect SCID mice suggests that MCP5 may be involved in evading one or more of these innate immune defenses still active in SCID mice. By contrast, NSG mice possess the *scid* mutation along with a targeted mutation in the IL2rg gene. This mutation not only results in a lack of T and B cells but also in the absence of functional NK cells and impairs various other innate immune responses, including cytokine production, macrophage phagocytosis, dendritic cell antigen presentation, and the complement system [70]. The observation that the *mcp5* mutant can infect NSG mice suggests that the absence of NK cells, along with resulting innate immune deficiencies, permits the *mcp5* mutant to establish infection.

Several lines of evidence suggest that the *mcp5* mutant’s inability to establish infection is linked to increased NK cell activation. First, NK cell depletion in both C3H and SCID mice significantly enhanced the infectivity of the *mcp5* mutant (**Fig 10**). NK cells are key producers of IFN-γ in response to *B*. *burgdorferi* infections [78,84,85]. Although previous studies have shown that NK cell depletion does not affect bacterial burdens in joint tissues and the severity of Lyme arthritis in mice, the impact of NK cells on spirochete burdens in other tissues, such as the skin during acute infection, has not been fully examined [85,86]. Our data revealed a marked increase in spirochete loads for both wild-type *B*. *burgdorferi* and the *mcp5* mutant at the inoculation site following NK cell depletion. Second, in vitro co-culture assays demonstrated that the mcp5 mutant led to enhanced NK cell activation (**Fig 11**). NK cell activation is known to promote the recruitment of immune cells primarily through IFN-γ production [73–75]. Consistent with this, we observed reduced recruitment of macrophages, dendritic cells, and CD8+ T cells to the infection site in NK cell-depleted mice, suggesting that these cells play a crucial role in clearing the mcp5 mutant (**Fig 12**). Macrophages and dendritic cells are essential for recognizing and phagocytosing *B*. *burgdorferi* [87]. Interestingly, NK cell depletion resulted in an increase in neutrophils, suggesting that neutrophils may not play a primary role in clearing the mcp5 mutant; rather, their increase likely reflects a compensatory response to alterations in the immune environment. A key question remains: how does the *mcp5* mutant enhance NK cell activation by infected macrophages? One possibility is that macrophages phagocytose the *mcp5* mutant more efficiently than wild-type spirochetes. The exact mechanism by which mutant-infected macrophages lead to enhanced NK cell activation remains to be elucidated. Nonetheless, our findings support the hypothesis that MCP5 is important for *B*. *burgdorferi* to evade the innate immune response.

How does MCP5 facilitate evasion of the host innate immune response? As a member of the methyl-accepting chemotaxis protein family, MCP5 is predicted to function as a receptor that binds ligands directly or interacts with ligand-binding proteins, transducing signals to downstream signaling proteins to mediate chemotaxis, guiding spirochetes to move toward higher concentrations of attractants or away from repellents [88]. Several chemo-attractants of *B*. *burgdorferi* have been identified, including serum, glucosamine, N-acetylglucosamine (NAG), glutamate, tick salivary gland, and tick salivary gland protein Salp12 [16,48–50]. MCP5 is highly expressed *in vitro*; but the *mcp5* mutant has a normal swimming behavior and still responds to rabbit serum and NAG (**S1 Fig**), suggesting that MCP5 is not chemotactic to these known chemoattractants *in vitro*. Given that the *mcp5* mutant could establish infection and disseminate in NSG mice but the chemotactic mutant, the *cheA2* mutant, couldn’t (**Fig 9C** and **Table 2**), it suggests that the *mcp5* mutant has normal chemotactic function in NSG mice. Thus, MCP5-associated chemotaxis is specific to the innate immune responses present in C3H and SCID mice. It is highly plausible that MCP5 senses yet-to-be-identified host signals, aiding in the evasion of the innate immune response. In this regard, structural modeling analyses revealed that the N-terminus of MCP5 likely contains a double-Cache (dCache) domain (**Fig 1**), which is found in the most abundant extracellular sensor superfamily in prokaryotes. The N-terminal Caches domain often binds ligands in other receptors. However, the most N-terminal Cache domain of MCP5 has relatively low sequence homology to domains of known structure, and the 3D prediction does not fully conform to the Cache fold(**Fig 1**). Despite this, we tested several potential ligands of Cache domains, including deoxy- and ribonucleosides (e.g., adenosine, cytidine, uridine, and pyrimidine) and sugars (e.g., D-ribose and pyruvate) but no significant difference between the wild type and the *mcp5* mutant in sensing these compounds. Thus, it is highly plausible that MCP5 senses yet-to-be-identified host signals, aiding in the evasion of the innate immune response. Nonetheless, this study demonstrates that MCP5 plays an essential role in the enzootic cycle of *B*. *burgdorferi*. These findings lay the groundwork for further elucidation of how *B*. *burgdorferi* utilizes MCP-mediated chemotaxis and motility to navigate between and within the tick vector and the vertebrate host.

## Materials and methods

### Ethics statement

All animal experiments were approved by the IACUC of Indiana University School of Medicine under protocol number # 20126. All experiments were in accordance with the institutional guidelines.

### *B*. *burgdorferi* strains and culture conditions

Low-passage, virulent *B*. *burgdorferi* strain B31 was used in this study [89]. Spirochetes were cultivated in Barbour-Stoenner-Kelly (BSK-II) medium supplemented with 6% rabbit serum (Pel-Freez Biologicals, Rogers, AR) [78] at 37°C with 5% CO_2_. At the time of growth, appropriate antibiotics were added to the cultures with the following final concentrations: 300 μg/ml for kanamycin and 50 μg/ml for streptomycin. The constructed suicide vectors for inactivation (pYZ001) and complementation (pYZ006) were maintained in *Escherichia coli* strain DH5α. The antibiotic concentrations used for *E*. *coli* selection were as follows: kanamycin (50 μg/ml) and streptomycin (50 μg/ml). A list of all the *B*. *burgdorferi* strains and plasmids used in the present study are represented in **S1 Table**.

### Cell lines

The human NK cell line, NK-92 was purchased from ATCC (CRL-2407) and maintained in MyeloCult H5100 media (StemCell Technologies, 05150) containing IL-2 (20 ng/ml, Miltenyi, 130-097-744) and Horse Serum (12.6%, Gibco, 16050122). The human THP-1 cell line was used from our lab cell lines inventory and was maintained in RPMI 1640 (Corning, 10-041-CV) supplemented with 10% heat-inactivated FBS (Fetal Bovine Serum), 100U/ml penicillin, 100 μg/ml streptomycin, and 25 mM HEPES.

### Immunoblot analysis

Spirochetes from various stages of growth were harvested by centrifuging at 8,000 × g for 10 min, followed by washing with PBS three times (pH 7.4) at 4°C. Cell pellets were suspended in SDS buffer containing 50 mM Tris-HCl (pH 8.0), 0.3% sodium dodecyl sulfate (SDS) and 10 mM dithiothreitol (DTT). Cell lysates (10^8^ cells per lane) were separated by 12% SDS-polyacrylamide gel electrophoresis (PAGE) and transferred to nitrocellulose membranes (GE-Healthcare, Milwaukee, WI). Membranes were blotted with rat polyclonal antibody against MCP5 (1:3,000 dilution) and monoclonal antibody against FlaB (1:1,000 dilution), followed by goat anti-rat lgG-HRP secondary antibody (1:1,000; Santa Cruz Biotechnology). Detection of horseradish peroxidase activity was determined using the enhanced chemiluminescence method (Thermo Pierce ECL Western Blotting Substrate) with subsequent exposure to X-ray films.

### AlphaFold model generation and analysis

To build models for *B*. *burgdorferi* (Bb) MCPs, protein sequences for *B*. *burgdorferi* MCP1 (O51525), MCP2 (O51542), MCP3 (O51543), MCP4 (O51623) and MCP5 (O51624) were retrieved from the UniProt database and submitted for automated model-building using AlphaFold2 [60]. All parameters were kept at their default values for model building.

### Generation of *mcp5* deletion mutant and its isogenic complemented strain

To inactivate *mcp5* in *B*. *burgdorferi* strain B31, a suicide vector pYZ001 was constructed. The regions of DNA corresponding to 1.5 kb upstream and downstream of *mcp5* were PCR amplified using specific set of primers PRYZ001/PRYZ002 (up) and PRYZ003/PRYZ004 (downstream) from *B*. *burgdorferi* (see S2 Table). The PCR products were cloned into a suicide plasmid pUC-Kan containing a *flaB* promoter driven kanamycin marker (*kan*) at ApaI and XbaI restriction sites (for upstream fragment) and HindIII and BamHI sites (for downstream fragment), respectively. The resulting suicidal plasmid pYZ001 was transformed into wild-type *B*. *burgdorferi* B31 as previously reported [68], and positive clones were selected based on kanamycin resistance and further validated by PCR and Western blot analyses. Endogenous plasmid profiles were performed for the *mcp5* mutant clones as previously described [66,90].

For *cis* complementation (gene replacement), the suicidal plasmid pYZ006 was generated as follows. The regions containing the full length *mcp5* were PCR amplified using specific sets of primers PRYZ009/PRYZ010 (upstream fragment) and PRYZ011/PRYZ012 (downstream fragment) from *B*. *burgdorferi* genomic DNA (see **S2 Table**). The upstream fragments were then cloned into the suicide vector pCT007 at ApaI and SalI restriction sites and XmaI and XbaI sites, respectively. The resulting suicidal plasmid pYZ006 was transformed into the *mcp5* mutant, and streptomycin resistant and kanamycin sensitive clones were selected, confirmed by PCR and Western blot analyses.

### 5′ Rapid amplification of cDNA end (5′ RACE) analysis

This assay was conducted as previously described [91]. In brief, wild-type *B*. *burgdorferi* B31 A3-68 cells were cultivated at 37°/pH 7.5 until late log phase and then harvested for RNA extraction using NucleoSpin RNA kit, following the manufacturer’s instruction (Macherey-Nagel, Bethlehem, PA). 5′ RACE was carried out using SMARTer RACE 5′/3′ Kit (Takara Bio USA, Mountain View, CA) to identify the transcription start site (TSS) of *mcp4* and *mcp5* genes following the manufacturer’s protocol. Primers used for the 5’RACE (Primers) were listed in S2 Table.

### *B*. *burgdorferi* motility and chemotaxis assays

Bacterial cell motility (wild-type *B*. *burgdorferi* B31M, the *mcp5* mutant, the *mcp5* complemented strain) was measured using a computer-based motion tracking system as previously described [48]. The *flaB* mutant (*flaB*^*-*^), a previously constructed non-motile mutant [92], served as a negative control. Briefly, late-log phase *B*. *burgdorferi* cultures were first diluted (1:1) in BSK-II medium and then 10 μl of the diluted cultures were mixed with an equal volume of 2% methylcellulose, and then subjected to dark-field microscopy. Spirochetes were video captured with iMovie software on a Mac computer and then exported as QuickTime movies, which were further imported into OpenLab (Improvision Inc., Coventry, UK) where the frames were cropped, calibrated, and saved as LIFF files. The software package Velocity (Improvision Inc.) was used to track individual moving cells to measure their velocities. For each bacterial strain, at least 20 cells were recorded for up to 30 sec. The average cell swimming velocities (μm/s) of tracked cells were calculated. Swimming plate assays were performed using 0.35% agarose with BSK-II medium diluted 1:10 with Dulbecco’s phosphate-buffered saline (DPBS, pH 7.5), as previously described [23,24,92]. The plates were incubated for 4–5 days at 34°C in the presence of 3.4% CO_2_. The diameters of swim rings were measured and recorded in millimeters (mm). The average diameters of each strain were calculated from four independent plates. Capillary tube assays were carried out as previously documented with minor modifications [48]. In brief, *B*. *burgdorferi* cells were grown to late-log phase (~5–7 × 10^7^ cells/ml) and harvested by low-speed centrifugations (1,800 × *g*). The harvested cells were then resuspended in the motility buffer. Capillary tubes filled with either the attractant (0.1 M N-acetylglucosamine dissolved in the motility buffer, or 0.5% rabbit serum) or only motility buffer (negative control) were sealed and inserted into microcentrifuge tubes containing 200 μl of resuspended cells (7 × 10^8^ cells/ml). After 2 hrs incubation at 34°C, the solutions were expelled from the capillary tubes, and spirochete cells were enumerated using Petroff-Hausser counting chambers under a dark-field microscope. A positive chemotactic response was defined as at least twice as many cells entering the attractant-filled tubes as the buffer-filled tubes. For the tracking, swimming plate, and capillary assays, the results are expressed as means ± standard errors of the means (SEM). The significance of the difference between different strains was evaluated with an unpaired Student *t* test (*P* value < 0.01).

### Mouse infection studies

Four-week-old C3H/HeN mice, C3H/SCID and NSG mice (Harlan, Indianapolis, IN) were subcutaneously inoculated with two doses of spirochetes (1×10^5^ and 1×10^6^) respectively. Ear punch biopsy samples were taken at 2- and 3-week post-injection. At 4 weeks post-injection, mice were euthanized, and multiple tissues (i.e., ear, joint, heart, skin and bladder tissues from each mouse) were harvested. All tissues were cultivated in 2 ml of the BSK-II medium (Sigma-Aldrich, St. Louis, MO) containing an antibiotic mixture of phosphomycin (2 mg/ml), rifampin (5 mg/ml), and amphotericin B (250 mg/ml) (Sigma-Aldrich) to inhibit bacterial and fungal contamination. All cultures were maintained at 37°C and examined for the presence of spirochetes by dark-field microscopy beginning from 5 days after inoculation. A single growth-positive culture was used as the criterion to determine positive mouse infection.

### qRT-PCR analyses

For identifying the expression pattern of *mcp5 in vitro*, wild-type *B*. *burgdorferi* strain B31 was cultured in BSK-II medium at various conditions. RNA samples were extracted from *B*. *burgdorferi* cultures using the RNeasy mini kit (Qiagen, Vanelcia, CA) according to the manufacturer’s protocols, followed by an on-column Digestion RNase-free DNase I treatment (Promega, Madison, WI). Quality of the isolated RNA was confirmed using PCR amplification of *B*. *burgdorferi flaB* (to check for DNA contamination). The cDNA was synthesized using the SuperScript III reverse transcriptase with random primers (Invitrogen, Carlsbad, CA). Quantitative PCR (qPCR) was performed in triplicate on an QuantStudio thermocycler. Calculations of relative levels of transcript were normalized with *flaB* transcript levels as previous reported [93].

For quantifying *mcp5* and *ospC* expression in infected mice, four-week-old C3H/HeN mice were injected with wild-type *B*. *burgdorferi* strain B31M at a dose of 1×10^4^ spirochetes per mouse. Mice were euthanized at different time points as indicated and mouse tissues were harvested and homogenized using the FastPrep-24 (MP Biomedicals). Total RNA was isolated using the TRIzol reagent (Thermo Fisher Scientific) according to the manufacturer’s instructions. To eliminate DNA contamination, samples were further digested with RNase-free DNaseI (Qiagen), purified using the RNeasy mini kit (Qiagen) and analyzed with NanoDrop Spectrophotometer (Thermo Fisher Scientific). cDNA was synthesized using the PrimeScript 1st strand cDNA Synthesis Kit (Takara Bio USA). For RNA analysis of spirochetes in ticks, 10 groups of fed larvae (3 ticks per group), 3 groups of unfed nymphs (40 ticks per group), and 10 groups of fed nymphs (one tick per data point) were used. Given the low levels of bacterial RNA in mouse tissues, the specific primers for each gene target were used for cDNA synthesis instead of random primers. To quantify the transcript levels of genes of interest, an absolute quantitation method was used to create a standard curve for the qRT-PCR assay according to the manufacturer’s protocol (Strategene, La Jolla, CA). Briefly, the PCR product of the *flaB* gene served as a standard template. A series of tenfold dilutions (10^2^-10^7^copies/ml) of the standard template was prepared, and qRT-PCR was performed to generate a standard curve by plotting the initial template quantity against the Ct values for the standards. The quantity of the targeted genes in the cDNA samples was calculated using their Ct values and the standard curve. The samples were assayed in triplicate using the ABI 7000 Sequence Detection System and PowerUp SYBR Green Master Mix (Applied Biosystems). The levels of the target gene transcript were reported as per 1000 copies of *flaB*. The primers used for the qRT-PCR analysis are depicted in S2 Table.

### Microinjection of *B*. *burgdorferi* into nymphal ticks

Microinjection and tick-mouse experiments were approved by the IACUC of Indiana University School of Medicine under protocol number #11339. *I*. *scapularis* nymphs were obtained from the Tick Rearing Facility at Oklahoma State University (Stillwater, OK). Microinjection was used to introduce spirochetes into the gut of nymphs as previously described [68]. Briefly, each *B*. *burgdorferi* strain was cultivated under normal conditions in BSK-II medium in the presence of corresponding selective antibiotics. Spirochetes were harvested by centrifugation and concentrated in BSK-II to a density of 5 × 10^8^ spirochetes/ml. A total of 10 μl of the cell suspension was then loaded into a 1mm diameter glass capillary needle (World Precision Instruments Inc.) by using a micro loader (Eppendorf AG). The bacterial suspension was then injected into the rectal aperture of unfed nymphal ticks by using a FemtoJet microinjector system (Eppendorf AG) as previously described [68].

### Assessment of spirochete transmission to mice by encapsulated nymphs

Transmission of spirochetes from *I*. *scapularis* ticks to C3H/HeN mice was assessed using artificially infected nymphs via microinjection as described above. Mice were anesthetized, infected ticks were confined to a capsule affixed to the shaved back of a naive C3H/HeN mouse (9 ticks per mouse). The ticks were allowed to feed to repletion (3 to 5 days) and then collected for DNA extraction. Subsequently, each sample of tick DNA was used to determine bacterial burdens by qPCR. Infected mice were then subjected to qPCR analysis to assess spirochetal burden in mouse tissues or culturing for *Borrelia* growth.

### Extraction of tick DNA

DNA was isolated from engorged nymphs using the DNeasy Blood & Tissue Kits (QIAGEN) according to the manufacturer’s instructions. Spirochete burdens within infected ticks were assessed with primer pairs of q-*flaB*-F/R and q-*Tactin*-F/R (see **S2 Table**). Absolute copy numbers of *flaB* are quantified as spirochete loads in ticks.

### NK cell Depletion

To deplete NK cells, C3H/HeN and SCID mice were administrated rabbit polyclonal anti-asialo GM1 antibody (BioLegend) as described previously [86]. Groups of age-matched mice received intraperitoneal (i.p) injections of 50 μl (1.8 mg) anti-asialo GM1 on the day of infection (day 0) and again on day 4 after infection. NK cell depletions in the spleen were assessed by flow cytometry using the PE-Cy7-conjugated anti-CD49b (pan-NK cell antibody) and BV650-conjugated anti-CD3.

### Multicolor Flow cytometry

To confirm NK cell depletion, spleens from NK cell-depleted and non-depleted C3H mice were harvested and passed through a 70 μm cell strainer to generate single-cell suspensions. Red blood cells were lysed using RBC lysis buffer and resuspended in FACS buffer (2% FBS in PBS). To elucidate the recruitment of immune cells at the site of infection, the skin was harvested, digested with Liberase TL (100μg/ml, Sigma, 5401020001) and DNase I (10μg/ml, Sigma, 11284932001) in RPMI with 5% FBS for 60 minutes at 37°C, and passed through 70 μm strainers. The resulting cells were washed, resuspended in 40% Percoll (Cytiva, 17089102), and centrifuged at 2,000 rpm for 10 minutes. The supernatant was discarded, and cell pellets were resuspended in the FACS buffer. The single cells generated from both the spleen and skin were counted using Trypan blue exclusion and stained with LIVE/DEAD dye (Fixable viability Dye eFluor 780, Invitrogen), followed by blocking with CD16/32 (BD Pharmingen, 553142) for 15 minutes at 4°C. Spleen cells were stained with the anti-**CD45 (clone:30-F11,103133), -CD3 (clone:17A2, 100229),** and **-CD49b (clone:DX5,108922) Abs while skin single cells were stained with the anti-CD45 (clone:30-F11,103133), -CD3 (clone:17A2, 100229), -CD4 (clone:GK1.5, 100410), -CD8a (clone:53–6.7, 100750), -CD11b (clone:M1/70, 101215), -F4/80 (clone:BM8, 123109), -Ly6C (clone:HK1.4, 128049), -Ly6G (clone:1A8, 127613), -CD11c (clone:N418, 117327),** and **-**I-A/I-E (clone:M5/114.15.2, 107605) Abs for 30 minutes at 4°C in the dark. All the antibodies were purchased from BioLegend. After staining, cells were washed, fixed in **1% PFA (Paraformaldehyde)**, and subjected to **flow cytometry analysis (Becton Dickenson)**. **Single-stained and unstained controls** were used to set gating parameters and compensation. The data were analyzed using FlowJo software (version 10, BD Life Sciences).

### NK cell activation Assay

THP-1 cells were differentiated into macrophage-like cells by treatment with phorbol 12-myristate 13-acetate (PMA) at a concentration of 5 ng/ml for 24 hours. Following differentiation, the THP-1 cells (1*10^5 cells/well) were infected with WT, Δ*mcp5*, or *mcp5*^*com*^ strains of *B*. *burgdorferi* at a multiplicity of infection (MOI) of 10 for 6 hours. THP-1 cells were then washed three times with cold PBS to remove extracellular *B*. *burgdorferi*. Subsequently, NK-92 cells were added to the culture at a 1:1 ratio (1*10^5 cells/well). After 24 hours of co-culture, supernatants were collected, and IFN-γ levels were determined by ELISA (BioLegend, 430104) following the manufacturer’s instructions. THP-1 and NK-92 cells cultured alone, with and without *B*. *burgdorferi* infection, were used as controls.

## Supporting information

S1 Table List of *B*. *burgdorferi* strains and plasmids used in present study. (DOCX)

S2 Table List of primers used in present study. (DOCX)

S1 Fig The *mcp5* mutant has no defect in motility and chemotaxis *in vitro*. (**A**) Swimming plate assay. Swimming plate assays for wild-type *B*. *burgdorferi* B31M, the *mcp5* mutant, and the *mcp5* complemented strain were performed using 0.35% agarose with BSK-II medium diluted 1:10 with DPBS. The diameters of the swim rings were measured, and average diameters of each strain were calculated from four independent plates. The *flaB* mutant (Δ*flaB*) served as a negative control. (**B**) Motion tracking analysis. Spirochetes were video captured using a computer-based motion tracking system. The average cell swimming velocities (μm/s) of tracked cells were calculated. (**C**) Capillary chemotaxis assays. *B*. *burgdorferi* cells were resuspended in the motility buffer and subjected to capillary assays for chemotaxis to acetylglucosamine and rabbit serum. Spirochetes were enumerated using Petroff-Hausser counting chambers under a dark-field microscope. For the tracking, swimming plate, and capillary assays, the results are expressed as means ± standard errors of the means (SEM). The significance of the difference between different strains was evaluated with an unpaired Student *t* test (*P* value < 0.01).
(DOCX)

S2 Fig Representative gating strategy to assess NK cell depletion in C3H mice. Spleens from C3H/HeN mice of untreated or treated with anti-Asiola-GM1 blocking antibody were harvested, and single-cell suspensions were prepared as outlined in the Materials and Methods. Cells were stained with antibodies against CD45, CD3, and CD49b. The gating strategy shown identifies NK cell populations (CD45+CD3-CD49b+) by excluding debris, focusing on single, live cells. Single-stained and unstained controls were used to define gating parameters and ensure accurate compensation.
(DOCX)

S3 Fig Representative gating strategy to assess NK cell depletion in SCID mice. Spleens from SCID mice of untreated or treated with anti-Asiola-GM1 blocking antibody were harvested, and single-cell suspensions were prepared as outlined in the Materials and Methods. Cells were stained with antibodies against CD45, CD3, and CD49b. The gating strategy shown identifies NK cell populations (CD45+CD3-CD49b+) by excluding debris, focusing on single, live cells. Single-stained and unstained controls were used to define gating parameters and ensure accurate compensation.
(DOCX)

S4 Fig Gating strategy to assess immune cell infiltration at the site of infection. Skin cells from wild-type or NK cell-depleted C3H mice (n = 4) challenged with the *mcp5* mutant (1 × 10^5/mouse) were harvested and dissociated as described in Materials and Methods. Cells were stained with antibodies against CD45, CD3, CD4, CD8a, CD11b, F4/80, CD11c, and I-A/I-E. The outlined gating strategy shows the approach used to identify the following populations, excluding debris and gating on single cells: live cells, CD45+ cells, CD45+CD3+CD4+ T cells, CD45+CD3+CD8+ T cells, CD45+CD11b+F4/80+I-A/I-E+ macrophages, CD45+CD11c+I-A/I-E+ dendritic cells, and CD45+CD11b+Ly6G+ neutrophils. Single-stained and unstained controls were used to set gating parameters and ensure proper compensation.
(DOCX)

## References

[1] Mattingly H, Kamino K, Machta B, Emonet T. *Escherichia coli* chemotaxis is information limited. Nat Phys. 2021;17(12):1426–31. 35035514 10.1038/s41567-021-01380-3 PMC8758097

[2] Wadhams GH, Armitage JP. Making sense of it all: bacterial chemotaxis. Nat Rev Mol Cell Biol. 2004;5(12):1024–37. doi: 10.1038/nrm1524 15573139

[3] Webre DJ, Wolanin PM, Stock JB. Bacterial chemotaxis. Curr Biol. 2003;13(2):R47–R9. doi: 10.1016/s0960-9822(02)01424-0 12546801

[4] Alexander RP, Lowenthal AC, Harshey RM, Ottemann KM. CheV: CheW-like coupling proteins at the core of the chemotaxis signaling network. Trends Microbiol. 2010;18(11):494–503. doi: 10.1016/j.tim.2010.07.004 20832320 PMC2975053

[5] Sourjik V. Receptor clustering and signal processing in Escherichia coli chemotaxis. Trends Microbiol. 2004;12(12):569–76. 15539117 10.1016/j.tim.2004.10.003

[6] Salah Ud-Din AIM, Roujeinikova A. Methyl-accepting chemotaxis proteins: a core sensing element in prokaryotes and archaea. CELL MOL LIFE SCI 2017;74:3293–303. doi: 10.1007/s00018-017-2514-0 28409190 PMC11107704

[7] Collins KD, Lacal J, Ottemann KM. Internal sense of direction: sensing and signaling from cytoplasmic chemoreceptors. Microbiol Mol Biol Rev. 2014;78(4):672–84. Epub 2014/11/28. doi: 10.1128/MMBR.00033-14 ; PubMed Central PMCID: PMC4248653. 25428939 PMC4248653

[8] Gestwicki JE, Lamanna AC, Harshey RM, McCarter LL, Kiessling LL, Adler J. Evolutionary conservation of methyl-accepting chemotaxis protein location in Bacteria and Archaea. J Bacteriol. 2000;182(22):6499–502. doi: 10.1128/JB.182.22.6499-6502.2000 11053396 PMC94798

[9] Wadhams GH, Martin AC, Armitage JP. Identification and localization of a methyl-accepting chemotaxis protein in *Rhodobacter sphaeroides*. Mol Microbiol. 2000;36(6):1222–33. 10931275 10.1046/j.1365-2958.2000.01936.x

[10] Hickman JW, Tifrea DF, Harwood CS. A chemosensory system that regulates biofilm formation through modulation of cyclic diguanylate levels. Proc Natl Acad Sci U S A. 2005;102(40):14422–7. doi: 10.1073/pnas.0507170102 16186483 PMC1234902

[11] Berleman JE, Bauer CE. Involvement of a Che-like signal transduction cascade in regulating cyst cell development in *Rhodospirillum centenum*. Mol Microbiol. 2005;56(6):1457–66. doi: 10.1111/j.1365-2958.2005.04646.x 15916598

[12] Luu RA, Kootstra JD, Nesteryuk V, Brunton CN, Parales JV, Ditty JL, et al. Integration of chemotaxis, transport and catabolism in *Pseudomonas putida* and identification of the aromatic acid chemoreceptor PcaY. Mol Microbiol. 2015;96(1):134–47. 25582673 10.1111/mmi.12929

[13] Harkey CW, Everiss KD, Peterson KM. The *Vibrio cholerae* toxin-coregulated-pilus gene tcpI encodes a homolog of methyl-accepting chemotaxis proteins. Infect Immun. 1994;62(7):2669–78. 8005659 10.1128/iai.62.7.2669-2678.1994 PMC302867

[14] Steere AC, Strle F, Wormser GP, Hu LT, Branda JA, Hovius JW, et al. Lyme borreliosis. Nat Rev Dis Primers. 2016;2(1):1–19. doi: 10.1038/nrdp.2016.90 27976670 PMC5539539

[15] Radolf JD, Caimano MJ, Stevenson B, Hu LT. Of ticks, mice and men: understanding the dual-host lifestyle of Lyme disease spirochaetes. Nat Rev Micro. 2012;10(2):87–99. doi: 10.1038/nrmicro2714 22230951 PMC3313462

[16] Murfin KE, Kleinbard R, Aydin M, Salazar SA, Fikrig E. *Borrelia burgdorferi* chemotaxis toward tick protein Salp12 contributes to acquisition. Ticks Tick Borne Dis. 2019;10(5):1124–34. Epub 2019/06/18. doi: 10.1016/j.ttbdis.2019.06.002 ; PubMed Central PMCID: PMC7792743. 31204044 PMC7792743

[17] Piesman J, Mather T, Sinsky R, Spielman A. Duration of tick attachment and *Borrelia burgdorferi* transmission. J Clin Microbiol. 1987;25(3):557–8. 3571459 10.1128/jcm.25.3.557-558.1987 PMC265989

[18] Kurokawa C, Lynn GE, Pedra JHF, Pal U, Narasimhan S, Fikrig E. Interactions between *Borrelia burgdorferi* and ticks. Nat Rev Microbiol. 2020;18(10):587–600. doi: 10.1038/s41579-020-0400-5 32651470 PMC7351536

[19] Charon NW, Goldstein SF. Genetics of motility and chemotaxis of a fascinating group of bacteria: the spirochetes. Annu Rev Genet. 2002;36:47. doi: 10.1146/annurev.genet.36.041602.134359 12429686

[20] Dunham-Ems SM, Caimano MJ, Pal U, Wolgemuth CW, Eggers CH, Balic A, et al. Live imaging reveals a biphasic mode of dissemination of *Borrelia burgdorferi* within ticks. J CLIN INVEST. 2009;119(12):3652–65. 19920352 10.1172/JCI39401 PMC2786795

[21] Motaleb MA, Liu J, Wooten RM. Spirochetal motility and chemotaxis in the natural enzootic cycle and development of Lyme disease. CURR OPIN MICROBIOL. 2015;28:106–13. doi: 10.1016/j.mib.2015.09.006 26519910 PMC4688064

[22] Motaleb M, Sultan SZ, Miller MR, Li C, Charon NW. CheY3 of *Borrelia burgdorferi* is the key response regulator essential for chemotaxis and forms a long-lived phosphorylated intermediate. J Bacteriol. 2011;193(13):3332–41. 21531807 10.1128/JB.00362-11 PMC3133270

[23] Li C, Bakker RG, Motaleb MA, Sartakova ML, Cabello FC, Charon NW. Asymmetrical flagellar rotation in *Borrelia burgdorferi* nonchemotactic mutants. Proc Natl Acad Sci U S A. 2002;99(9):6169–74. 11983908 10.1073/pnas.092010499 PMC122921

[24] Zhang K, Liu J, Tu Y, Xu H, Charon NW, Li C. Two CheW coupling proteins are essential in a chemosensory pathway of *Borrelia burgdorferi*. Mol Microbiol. 2012;85(4):782–94. 22780444 10.1111/j.1365-2958.2012.08139.x PMC3418435

[25] Xu H, Raddi G, Liu J, Charon NW, Li C. Chemoreceptors and flagellar motors are subterminally located in close proximity at the two cell poles in spirochetes. J Bacteriol. 2011;193(10):2652–6. doi: 10.1128/JB.01530-10 21441520 PMC3133147

[26] Zhang K, Liu J, Charon NW, Li C. Hypothetical protein BB0569 is essential for chemotaxis of the Lyme disease spirochete *Borrelia burgdorferi*. J Bacteriol. 2016;198(4):664–72. 10.1128/JB.00877-15 PMC4751812 26644432

[27] Fraser CM, Casjens S, Huang WM, Sutton GG, Clayton R, Lathigra R, et al. Genomic sequence of a Lyme disease spirochaete, *Borrelia burgdorferi*. Nat. 1997;390(6660):580–6. 10.1038/37551 9403685

[28] Sze CW XH, Motaleb MA, Wolgemuth CW, Liu J, Charon NW, Li C. Dancing with the Star: Borrelia burgdorferi, a Solo Dancer with All the Right Moves. In: JD R DS S, editors. Lyme Disease and Relapsing Fever Spirochetes: Genomics, Molecular Biology, Host Interactions and Disease Pathogenesis. U.K: Caister Academic Press 2021. p. 221–50.

[29] Samuels DS, Lybecker MC, Yang XF, Ouyang Z, Bourret TJ, Boyle WK, et al. Gene Regulation and Transcriptomics. Curr Issues Mol Biol. 2021;42:223–66. Epub 2020/12/11. doi: 10.21775/cimb.042.223 ; PubMed Central PMCID: PMC7946783. 33300497 PMC7946783

[30] Stevenson B. The Lyme disease spirochete, *Borrelia burgdorferi*, as a model vector-borne pathogen: insights on regulation of gene and protein expression. CURR OPIN MICROBIOL 2023;74:102332. doi: 10.1016/j.mib.2023.102332 37279610 PMC10524203

[31] Ye M, Zhou Y, Lou Y, Yang XF. Genome reduction of *Borrelia burgdorferi*: two TCS signaling pathways for two distinct host habitats. SCI CHINA LIFE SCI. 2016;59(1):19. doi: 10.1007/s11427-015-4996-z 26740104 PMC5846617

[32] Samuels DS. Gene regulation in *Borrelia burgdorferi*. Annu Rev Microbiol. 2011;65:479–99. Epub 2011/08/02. doi: 10.1146/annurev.micro.112408.134040 . 21801026

[33] Caimano MJ, Drecktrah D, Kung F, Samuels DS. Interaction of the Lyme disease spirochete with its tick vector. Cell Microbiol. 2016;18(7):919–27. doi: 10.1111/cmi.12609 27147446 PMC5067140

[34] He M, Ouyang Z, Troxell B, Xu H, Moh A, Piesman J, et al. Cyclic di-GMP is essential for the survival of the Lyme disease spirochete in ticks. PLoS Pathog. 2011;7(6):e1002133. doi: 10.1371/journal.ppat.1002133 21738477 PMC3128128

[35] Kostick JL, Szkotnicki LT, Rogers EA, Bocci P, Raffaelli N, Marconi RT. The diguanylate cyclase, Rrp1, regulates critical steps in the enzootic cycle of the Lyme disease spirochetes. Mol Microbiol. 2011;81(1):219–31. doi: 10.1111/j.1365-2958.2011.07687.x 21542866 PMC3124615

[36] Caimano MJ, Kenedy MR, Kairu T, Desrosiers DC, Harman M, Dunham-Ems S, et al. The hybrid histidine kinase Hk1 is part of a two-component system that is essential for survival of *Borrelia burgdorferi* in feeding *Ixodes scapularis* ticks. Infect Immun. 2011;79(8):3117–30. doi: 10.1128/iai.05136-11 21606185 PMC3147546

[37] Sze CW, Smith A, Choi YH, Yang X, Pal U, Yu A, et al. Study of the response regulator Rrp1 reveals its regulatory role in chitobiose utilization and virulence of *Borrelia burgdorferi*. Infect Immun. 2013;81(5):1775–87. doi: 10.1128/iai.00050-13 23478317 PMC3647990

[38] Caimano MJ, Dunham-Ems S, Allard AM, Cassera MB, Kenedy M, Radolf JD. Cyclic di-GMP modulates gene expression in Lyme disease spirochetes at the tick-mammal interface to promote spirochete survival during the blood meal and tick-to-mammal transmission. Infect Immun. 2015;83(8):3043–60. doi: 10.1128/IAI.00315-15 ; PubMed Central PMCID: PMC4496621. 25987708 PMC4496621

[39] Bontemps-Gallo S, Lawrence K, Gherardini FC. Two Different Virulence-Related Regulatory Pathways in *Borrelia burgdorferi* Are Directly Affected by Osmotic Fluxes in the Blood Meal of Feeding *Ixodes* Ticks. PLoS Pathogens. 2016;12(8):e1005791. doi: 10.1371/journal.ppat.1005791 27525653 PMC4985143

[40] Novak EA, Sultan SZ, Motaleb MA. The cyclic-di-GMP signaling pathway in the Lyme disease spirochete, *Borrelia burgdorferi*. Front Cell Infect Microbiol. 2014;4(56). doi: 10.3389/fcimb.2014.00056 24822172 PMC4013479

[41] Sultan SZ, Pitzer JE, Boquoi T, Hobbs G, Miller MR, Motaleb MA. Analysis of the HD-GYP domain cyclic dimeric GMP phosphodiesterase reveals a role in motility and the enzootic life cycle of *Borrelia burgdorferi*. Infect Immun. 2011;79(8):3273–83. doi: 10.1128/iai.05153-11 21670168 PMC3147568

[42] Sultan SZ, Pitzer JE, Miller MR, Motaleb MA. Analysis of a *Borrelia burgdorferi* phosphodiesterase demonstrates a role for cyclic-di-guanosine monophosphate in motility and virulence. Mol Microbiol. 2010;77(1):128–42. Epub 2010/05/07. doi: 10.1111/j.1365-2958.2010.07191.x [pii]. ; PubMed Central PMCID: PMC2907449. 20444101 PMC2907449

[43] Caimano MJ, Eggers CH, Gonzalez CA, Radolf JD. Alternate sigma factor RpoS is required for the in vivo-specific repression of *Borrelia burgdorferi* plasmid lp54-borne *ospA* and *lp6*.*6* genes. J Bacteriol. 2005;187(22):7845–52. 16267308 10.1128/JB.187.22.7845-7852.2005 PMC1280317

[44] Fisher MA, Grimm D, Henion AK, Elias AF, Stewart PE, Rosa PA, et al. *Borrelia burgdorferi* σ^54^ is required for mammalian infection and vector transmission but not for tick colonization. Proc Natl Acad Sci U S A. 2005;102(14):5162–7. 15743918 10.1073/pnas.0408536102 PMC555983

[45] Hübner A, Yang X, Nolen DM, Popova TG, Cabello FC, Norgard MV. Expression of *Borrelia burgdorferi* OspC and DbpA is controlled by a RpoN-RpoS regulatory pathway. Proc Natl Acad Sci U S A. 2001;98(22):12724–9. Epub 2001/10/25. doi: 10.1073/pnas.231442498 98/22/12724 [pii]. ; PubMed Central PMCID: PMC60121. 11675503 PMC60121

[46] Boardman BK, He M, Ouyang Z, Xu H, Pang X, Yang XF. Essential role of the response regulator Rrp2 in the infectious cycle of *Borrelia burgdorferi*. Infect Immun. 2008;76(9):3844–53. 18573895 10.1128/IAI.00467-08 PMC2519420

[47] Ouyang Z, Narasimhan S, Neelakanta G, Kumar M, Pal U, Fikrig E, et al. Activation of the RpoN-RpoS regulatory pathway during the enzootic life cycle of *Borrelia burgdorferi*. BMC Microbiol. 2012;12(1):44. doi: 10.1186/1471-2180-12-44 22443136 PMC3320556

[48] Bakker RG, Li C, Miller MR, Cunningham C, Charon NW. Identification of specific chemoattractants and genetic complementation of a *Borrelia burgdorferi* chemotaxis mutant: flow cytometry-based capillary tube chemotaxis assay. APPL ENVIRON MICROB. 2007;73(4):1180–8. 10.1128/AEM.01913-06 PMC1828676 17172459

[49] Shi W, Yang Z, Geng Y, Wolinsky LE, Lovett MA. Chemotaxis in *Borrelia burgdorferi*. J Bacteriol. 1998;180(2):231–5. Epub 1998/01/24. doi: 10.1128/jb.180.2.231–235.1998 ; PubMed Central PMCID: PMC106876. 9440510 PMC106876

[50] Shih CM, Chao LL, Yu CP. Chemotactic migration of the Lyme disease spirochete (*Borrelia burgdorferi*) to salivary gland extracts of vector ticks. Am J Trop Med Hyg. 2002;66(5):616–21. Epub 2002/08/31. doi: 10.4269/ajtmh.2002.66.616 . 12201601

[51] Rogers EA, Terekhova D, Zhang HM, Hovis KM, Schwartz I, Marconi RT. Rrp1, a cyclic-di-GMP-producing response regulator, is an important regulator of *Borrelia burgdorferi* core cellular functions. Mol Microbiol. 2009;71(6):1551–73. Epub 2009/02/13. doi: 10.1111/j.1365-2958.2009.06621.x ; PubMed Central PMCID: PMC2843504. 19210621 PMC2843504

[52] Caimano M, Iyer R, Eggers C, Gonzalez C, Morton E, Gilbert M, et al. Analysis of the RpoS regulon in *Borrelia burgdorferi* in response to mammalian host signals provides insight into RpoS function during the enzootic cycle. Mol Microbiol. 2007;65(5):1193–217. doi: 10.1111/j.1365-2958.2007.05860.x 17645733 PMC2967192

[53] Ojaimi C, Brooks C, Casjens S, Rosa P, Elias A, Barbour A, et al. Profiling of temperature-induced changes in *Borrelia burgdorferi* gene expression by using whole genome arrays. Infect Immun. 2003;71(4):1689–705. 12654782 10.1128/IAI.71.4.1689-1705.2003 PMC152086

[54] Revel AT, Talaat AM, Norgard MV. DNA microarray analysis of differential gene expression in *Borrelia burgdorferi*, the Lyme disease spirochete. Proc Natl Acad Sci U S A. 2002;99:1562–7. 11830671 10.1073/pnas.032667699 PMC122230

[55] Drecktrah D, Lybecker M, Popitsch N, Rescheneder P, Hall LS, Samuels DS. The *Borrelia burgdorferi* RelA/SpoT homolog and stringent response regulate survival in the tick vector and global gene expression during starvation. PLoS Pathog. 2015;11(9):e1005160. doi: 10.1371/journal.ppat.1005160 ; PubMed Central PMCID: PMC4570706. 26371761 PMC4570706

[56] Xu H, Raddi G, Liu J, Charon NW, Li C. Chemoreceptors and flagellar motors are subterminally located in close proximity at the two cell poles in spirochetes. J Bacteriol. 2011;193(10):2652–6. Epub 2011/03/29. doi: 10.1128/JB.01530-10 ; PubMed Central PMCID: PMC3133147. 21441520 PMC3133147

[57] Sweeney EG, Perkins A, Kallio K, James Remington S, Guillemin K. Structures of the ligand-binding domain of Helicobacter pylori chemoreceptor TlpA. Protein Sci. 2018;27(11):1961–8. Epub 2018/09/02. doi: 10.1002/pro.3503 ; PubMed Central PMCID: PMC6201720. 30171638 PMC6201720

[58] Brewster JL, McKellar JL, Finn TJ, Newman J, Peat TS, Gerth ML. Structural basis for ligand recognition by a Cache chemosensory domain that mediates carboxylate sensing in Pseudomonas syringae. Sci Rep. 2016;6:35198. Epub 2016/10/14. doi: 10.1038/srep35198 ; PubMed Central PMCID: PMC5062169. 27734909 PMC5062169

[59] Matilla MA, Velando F, Tajuelo A, Martin-Mora D, Xu W, Sourjik V, et al. Chemotaxis of the Human Pathogen Pseudomonas aeruginosa to the Neurotransmitter Acetylcholine. mBio. 2022;13(2):e0345821. Epub 2022/03/08. doi: 10.1128/mbio.03458-21 ; PubMed Central PMCID: PMC9040839. 35254130 PMC9040839

[60] Jumper J, Evans R, Pritzel A, Green T, Figurnov M, Ronneberger O, et al. Highly accurate protein structure prediction with AlphaFold. Nature. 2021;596(7873):583–9. Epub 2021/07/16. doi: 10.1038/s41586-021-03819-2 ; PubMed Central PMCID: PMC8371605 have filed non-provisional patent applications 16/701,070 and PCT/EP2020/084238, and provisional patent applications 63/107,362, 63/118,917, 63/118,918, 63/118,921 and 63/118,919, each in the name of DeepMind Technologies Limited, each pending, relating to machine learning for predicting protein structures. The other authors declare no competing interests. 34265844 PMC8371605

[61] Holm L, Laiho A, Toronen P, Salgado M. DALI shines a light on remote homologs: One hundred discoveries. Protein Sci. 2023;32(1):e4519. Epub 2022/11/25. doi: 10.1002/pro.4519 ; PubMed Central PMCID: PMC9793968. 36419248 PMC9793968

[62] Stevenson B, Schwan TG, Rosa PA. Temperature-related differential expression of antigens in the Lyme disease spirochete, *Borrelia burgdorferi*. Infect Immun. 1995;63:4535–9. 7591099 10.1128/iai.63.11.4535-4539.1995 PMC173648

[63] Yang X, Goldberg MS, Popova TG, Schoeler GB, Wikel SK, Hagman KE, et al. Interdependence of environmental factors influencing reciprocal patterns of gene expression in virulent *Borrelia burgdorferi*. Mol Microbiol. 2000;37:1470–9. 10998177 10.1046/j.1365-2958.2000.02104.x

[64] Carroll JA, Cordova RM, Garon CF. Identification of 11 pH-regulated genes in *Borrelia burgdorferi* localizing to linear plasmids. Infect Immun. 2000;68:6677–84. 11083781 10.1128/iai.68.12.6677-6684.2000 PMC97766

[65] Purser JE, Norris SJ. Correlation between plasmid content and infectivity in *Borrelia burgdorferi* Proc Natl Acad Sci U S A. 2000;97:13865–70. 11106398 10.1073/pnas.97.25.13865 PMC17667

[66] Bunikis I, Kutschan-Bunikis S, Bonde M, Bergström S. Multiplex PCR as a tool for validating plasmid content of *Borrelia burgdorferi*. J Microbiol Methods. 2011;86(2):243–7. doi: 10.1016/j.mimet.2011.05.004 21605603

[67] Casjens S, Palmer N, van Vugt R, Huang WM, Stevenson B, Rosa P, et al. A bacterial genome in flux: the twelve linear and nine circular extrachromosomal DNAs in an infectious isolate of the Lyme disease spirochete *Borrelia burgdorferi*. Mol Microbiol. 2000;35:490–516. 10672174 10.1046/j.1365-2958.2000.01698.x

[68] Yang XF, Pal U, Alani SM, Fikrig E, Norgard MV. Essential role for OspA/B in the life cycle of the Lyme disease spirochete. J Exp Med. 2004;199(5):641–8. doi: 10.1084/jem.20031960 14981112 PMC2213294

[69] Bosma GC, Custer RP, Bosma MJ. A severe combined immunodeficiency mutation in the mouse. Nat. 1983;301(5900):527–30. doi: 10.1038/301527a0 6823332

[70] Shultz LD, Lyons BL, Burzenski LM, Gott B, Chen X, Chaleff S, et al. Human lymphoid and myeloid cell development in NOD/LtSz-scid IL2R gamma null mice engrafted with mobilized human hemopoietic stem cells. J Immunol. 2005;174(10):6477–89. Epub 2005/05/10. doi: 10.4049/jimmunol.174.10.6477 . 15879151

[71] Cao X, Shores EW, Hu-Li J, Anver MR, Kelsall BL, Russell SM, et al. Defective lymphoid development in mice lacking expression of the common cytokine receptor gamma chain. Immunity. 1995;2(3):223–38. doi: 10.1016/1074-7613(95)90047-0 . 7697543

[72] Sze CW, Zhang K, Kariu T, Pal U, Li C. *Borrelia burgdorferi* needs chemotaxis to establish infection in mammals and to accomplish its enzootic cycle. Infect Immun. 2012;80(7):2485–92. Epub 2012/04/18. doi: 10.1128/iai.00145-12 ; PubMed Central PMCID: PMC3416460. 22508862 PMC3416460

[73] Björkström NK, Strunz B, Ljunggren H-G. Natural killer cells in antiviral immunity. Nature Reviews Immunology. 2022;22(2):112–23. doi: 10.1038/s41577-021-00558-3 34117484 PMC8194386

[74] Elemam NM, Ramakrishnan RK, Hundt JE, Halwani R, Maghazachi AA, Hamid Q. Innate Lymphoid Cells and Natural Killer Cells in Bacterial Infections: Function, Dysregulation, and Therapeutic Targets. Front Cell Infect Microbiol. 2021;11:733564. Epub 20211105. doi: 10.3389/fcimb.2021.733564 ; PubMed Central PMCID: PMC8602108. 34804991 PMC8602108

[75] Souza-Fonseca-Guimaraes F, Adib-Conquy M, Cavaillon JM. Natural killer (NK) cells in antibacterial innate immunity: angels or devils? Mol Med. 2012;18(1):270–85. Epub 20120330. doi: 10.2119/molmed.2011.00201 ; PubMed Central PMCID: PMC3324953. 22105606 PMC3324953

[76] Rogers EA, Terekhova D, Zhang H, Hovis KM, Schwartz I, Marconi RT. Rrp1, a cyclic-di-GMP-producing response regulator, is an important regulator of *Borrelia burgdorferi* core cellular functions. Mol Microbiol. 2009;71(6):1551–73. 19210621 10.1111/j.1365-2958.2009.06621.x PMC2843504

[77] Ouyang Z, Blevins JS, Norgard MV. Transcriptional interplay among the regulators Rrp2, RpoN and RpoS in *Borrelia burgdorferi*. Microbiol. 2008;154(9):2641–58. 10.1099/mic.0.2008/019992-0 18757798

[78] Oosting M, Brouwer M, Vrijmoeth HD, Pascual Domingo R, Greco A, ter Hofstede H, et al. *Borrelia burgdorferi* is strong inducer of IFN-γ production by human primary NK cells. Cytokine. 2022;155:155895. doi: 10.1016/j.cyto.2022.155895 35569383

[79] Sze CW, Smith A, Choi YH, Yang X, Pal U, Yu A, et al. Study of the response regulator Rrp1 reveals its regulatory role in chitobiose utilization and virulence of *Borrelia burgdorferi*. Infect Immun. 2013;81(5):1775–87. Epub 2013/03/13. doi: 10.1128/iai.00050-13 ; PubMed Central PMCID: PMC3647990. 23478317 PMC3647990

[80] Xu H, Sultan S, Yerke A, Moon KH, Wooten RM, Motaleb MA. *Borrelia burgdorferi* CheY2 is dispensable for chemotaxis or motility but crucial for the infectious life cycle of the spirochete. Infect Immun. 2017;85(1):e00264–16. 10.1128/IAI.00264-16 PMC5203640 27799336

[81] Sze CW, Zhang K, Lynch MJ, Iyer R, Crane BR, Schwartz I, et al. A chemosensory-like histidine kinase is dispensable for chemotaxis in vitro but regulates the virulence of *Borrelia burgdorferi* through modulating the stability of RpoS. PLOS Pathogens. 2023;19(11):e1011752. doi: 10.1371/journal.ppat.1011752 38011206 PMC10703414

[82] Novak EA, Sekar P, Xu H, Moon KH, Manne A, Wooten RM, et al. The *Borrelia burgdorferi* CheY3 response regulator is essential for chemotaxis and completion of its natural infection cycle. Cell Microbiol. 2016;18(12):1782–99. 27206578 10.1111/cmi.12617 PMC5116424

[83] Sultan SZ, Sekar P, Zhao X, Manne A, Liu J, Wooten RM, et al. Motor rotation is essential for the formation of the periplasmic flagellar ribbon, cellular morphology, and Borrelia burgdorferi persistence within Ixodes scapularis tick and murine hosts. Infect Immun. 2015;83(5):1765–77. Epub 2015/02/19. doi: 10.1128/IAI.03097-14 ; PubMed Central PMCID: PMC4399055. 25690096 PMC4399055

[84] Moore MW, Cruz AR, LaVake CJ, Marzo AL, Eggers CH, Salazar JC, et al. Phagocytosis of *Borrelia burgdorferi* and *Treponema pallidum* potentiates innate immune activation and induces gamma interferon production. Infect Immun. 2007;75(4):2046–62. Epub 20070112. doi: 10.1128/iai.01666-06 ; PubMed Central PMCID: PMC1865718. 17220323 PMC1865718

[85] Brown CR, Reiner SL. Activation of natural killer cells in arthritis-susceptible but not arthritis-resistant mouse strains following *Borrelia burgdorferi* infection. Infect Immun. 1998;66(11):5208–14. doi: 10.1128/iai.66.11.5208–5214.1998 ; PubMed Central PMCID: PMC108650. 9784524 PMC108650

[86] Miller JC, Ma Y, Bian J, Sheehan KC, Zachary JF, Weis JH, et al. A critical role for type I IFN in arthritis development following *Borrelia burgdorferi* infection of mice. J Immunol. 2008;181(12):8492–503. doi: 10.4049/jimmunol.181.12.8492 ; PubMed Central PMCID: PMC3024833. 19050267 PMC3024833

[87] Salazar JC, Pope CD, Sellati TJ, Feder HM, Jr., Kiely TG, Dardick KR, et al. Coevolution of markers of innate and adaptive immunity in skin and peripheral blood of patients with erythema migrans. J Immunol. 2003;171(5):2660–70. doi: 10.4049/jimmunol.171.5.2660 . 12928420

[88] Derr P, Boder E, Goulian M. Changing the Specificity of a Bacterial Chemoreceptor. J Mol Biol. 2006;355(5):923–32. doi: 10.1016/j.jmb.2005.11.025 16359703

[89] Barbour AG. Isolation and cultivation of Lyme disease spirochetes. Yale J Biol Med. 1984;57(4):521–5. Epub 1984/07/01. ; PubMed Central PMCID: PMC2589996. 6393604 PMC2589996

[90] Xiang X, Yang Y, Du J, Lin T, Chen T, Yang XF, et al. Investigation of ospC Expression Variation among *Borrelia burgdorferi* Strains. Front Cell Infect Microbiol. 2017;7:131. Epub 2017/05/06. doi: 10.3389/fcimb.2017.00131 ; PubMed Central PMCID: PMC5397415. 28473966 PMC5397415

[91] Lybecker MC, Samuels DS. Temperature-induced regulation of RpoS by a small RNA in *Borrelia burgdorferi*. MolMicrobiol. 2007;64(4):1075–89. 10.1111/j.1365-2958.2007.05716.x 17501929

[92] Motaleb MA, Corum L, Bono JL, Elias AF, Rosa P, Samuels DS, et al. *Borrelia burgdorferi* periplasmic flagella have both skeletal and motility functions. Proc Natl Acad Sci U S A. 2000;97(20):10899–904. 10995478 10.1073/pnas.200221797 PMC27121

[93] Chen T, Xiang X, Xu H, Zhang X, Zhou B, Yang Y, et al. LtpA, a CdnL-type CarD regulator, is important for the enzootic cycle of the Lyme disease pathogen. Emerg Microbes Infect. 2018;7(1):126–. doi: 10.1038/s41426-018-0122-1 . 29985409 PMC6037790

