# Supplementary material for: MCP5, a methyl-accepting chemotaxis protein regulated by both the Hk1-Rrp1 and Rrp2-RpoN-RpoS pathways, is required for the immune evasion of Borrelia burgdorferi
Source: PLoS Pathog. 2024 Dec 30;20(12):e1012327. doi: 10.1371/journal.ppat.1012327 (PMC11723614; doi:10.1371/journal.ppat.1012327)
Supplement: S1 Table — (DOCX) [file ppat.1012327.s001.docx]

**S1_Table.** **List of *B. burgdorferi* strains and plasmids used in present study.**

| Strains and plasmids used in the study | Description | Reference |
| --- | --- | --- |
| B31 | Wild-type *B. burgdorferi* | [1] |
| Δ*mcp5* | *mcp5* mutant *B. burgdorferi* (kanamycin^R^) | Present study |
| *mcp5^com^* | *mcp5* complement *B. burgdorferi* (streptomycin^R^) | Present study |
| Δ*flaB* | *flaB* mutant *B. burgdorferi* | [2] |
| Δ*rrp1* | *rrp1* mutant *B. burgdorferi* (streptomycin^R^) | [3] |
| *rrp2^G239C^* | *rrp2^G239C^* mutant *B. burgdorferi* (erythromycin^R^) | [4] |
| Δ*rpoN* | *rpoN* mutant *B. burgdorferi* (gentamycin^R^) | [5] |
| Δ*bosR* | *bosR* mutant *B. burgdorferi* (kanamycin^R^) | [6] |
| Δ*rpoS* | *rpoS* mutant *B. burgdorferi* (kanamycin^R^) | [7] |
| pYZ001 | *mcp5* knockout suicidal plasmid with (kanamycin^R^) | Present study |
| pYZ006 | *mcp5 c*omplementation suicidal plasmid with (streptomycin^R^) | Present study |

1. Barbour AG. Isolation and cultivation of Lyme disease spirochetes. Yale J Biol Med. 1984;57(4):521-5. Epub 1984/07/01. PubMed PMID: 6393604; PubMed Central PMCID: PMCPMC2589996.

2. Li C, Bakker RG, Motaleb MA, Sartakova ML, Cabello FC, Charon NW. Asymmetrical flagellar rotation in *Borrelia burgdorferi* nonchemotactic mutants. Proc Natl Acad Sci U S A. 2002;99(9):6169-74.

3. He M, Ouyang Z, Troxell B, Xu H, Moh A, Piesman J, et al. Cyclic di-GMP is essential for the survival of the Lyme disease spirochete in ticks. PLoS Pathog. 2011;7(6):e1002133. doi: 10.1371/journal.ppat.1002133.

4. Boardman BK, He M, Ouyang Z, Xu H, Pang X, Yang XF. Essential role of the response regulator Rrp2 in the infectious cycle of *Borrelia burgdorferi*. Infect Immun. 2008;76(9):3844-53.

5. Raghunandanan S, Priya R, Alanazi F, Lybecker MC, Schlax PJ, Yang XF. A Fur family protein BosR is a novel RNA-binding protein that controls rpoS RNA stability in the Lyme disease pathogen. Nucleic Acids Res. 2024:gkae114.

6. Ouyang Z, Kumar M, Kariu T, Haq S, Goldberg M, Pal U, et al. BosR (BB0647) governs virulence expression in *Borrelia burgdorferi*. Mol Microbiol. 2009;74(6):1331-43. doi: 10.1111/j.1365-2958.2009.06945.x.

7. Alanazi F, Raghunandanan S, Priya R, Yang XF. The Rrp2-RpoN-RpoS pathway plays an important role in the blood-brain barrier transmigration of the Lyme disease pathogen. Infect and Immun. 2023;91(11):e00227-23.
