# Supplementary material for: MCP5, a methyl-accepting chemotaxis protein regulated by both the Hk1-Rrp1 and Rrp2-RpoN-RpoS pathways, is required for the immune evasion of Borrelia burgdorferi
Source: PLoS Pathog. 2024 Dec 30;20(12):e1012327. doi: 10.1371/journal.ppat.1012327 (PMC11723614; doi:10.1371/journal.ppat.1012327)
Supplement: S2 Table — (DOCX) [file ppat.1012327.s002.docx]

**S2_Table. List of primers used in present study.**

| Primer | Sequence (5’-3’) | Purpose |
| --- | --- | --- |
| PRYZ001 | GATCGGGCCCTTGGCATGTTAAGTCTTCAG  GTTG | Generating pYZ001 |
| PRYZ002 | GATCTCTAGATTTTCAATAACTCCTTTTGCA  ACT | Generating pYZ001 |
| PRYZ003 | GATCAAGCTTGCAGATCAAAGCAAAGAATC  AGCA | Generating pYZ001 |
| PRYZ004 | GATCGGATCAAAATGGCAATACCCCAAGTC  CAGA | Generating pYZ001 |
| PRYZ009 | GATCGGGCCCTAGCTTTAAAGACGAATGAA  AATTC | Generating pYZ006 |
| PRYZ010 | GATCGTCGACCAATCTTCTACTTACTTTTCG  ATCT | Generating pYZ006 |
| PRYZ011 | GATCCCCGGGAAAAATCTTCTCTAACTTTTT  GGGC | Generating pYZ006 |
| PRYZ012 | GATCTCTAGAAAAATGGCAATACCCCAAGT  CCAGA | Generating pYZ006 |
| YZ-qPCR-flab-F2 | CACCAGCATCACTTTCAGGGTCTCA | *flaB* qPCR forward primer |
| YZ-qPCR-flab-R2 | TGTAGCAGGTGCTGGCTGTTGA | *flaB* qPCR reverse primer |
| qHXMactin-F2 | GCTGAGAGGGAAATCGTGCGTGAC | Mouse β-actin qPCR forward primer |
| qHXMactin-R2 | GGAGGAAGAGGATGCGGCAGTGGC | Mouse β-actin qPCR reverse primer |
| qHXTactin-F2 | CACACCGTCCCCATCTACGAAGG | Tick β-actin qPCR forward primer |
| qHXTactin-R2 | TCATCAGGTAGTCGGTCAGGTCC | Tick β-actin qPCR reverse primer |
| *mcp4* q-PCRFP | CTAGTGCATTGCAACAGGCA | *mcp4* qPCR forward primer |
| *mcp4* q-PCRRP | ATTCTTCAACGGCCCTACCA | *mcp4* qPCR reverse primer |
