# Supplementary material for: MCP5, a methyl-accepting chemotaxis protein regulated by both the Hk1-Rrp1 and Rrp2-RpoN-RpoS pathways, is required for the immune evasion of Borrelia burgdorferi
Source: PLoS Pathog. 2024 Dec 30;20(12):e1012327. doi: 10.1371/journal.ppat.1012327 (PMC11723614; doi:10.1371/journal.ppat.1012327)
Supplement: S1 Fig — (A) Swimming plate assay. Swimming plate assays for wild-type B. burgdorferi B31M, the mcp5 mutant, and the mcp5 complemented strain were performed using 0.35% agarose with BSK-II medium diluted 1:10 with DPBS. The diameters of the swim rings were measured, and average diameters of each strain were calculated from four independent plates. The flaB mutant (ΔflaB) served as a negative control. (B) Motion tracking analysis. Spirochetes were video captured using a computer-based motion tracking system. The average cell swimming velocities (μm/s) of tracked cells were calculated. (C) Capillary chemotaxis assays. B. burgdorferi cells were resuspended in the motility buffer and subjected to capillary assays for chemotaxis to acetylglucosamine and rabbit serum. Spirochetes were enumerated using Petroff-Hausser counting chambers under a dark-field microscope. For the tracking, swimming plate, and capillary assays, the results are expressed as means ± standard errors of the means (SEM). The significance of the difference between different strains was evaluated with an unpaired Student t test (P value < 0.01). (DOCX) [file ppat.1012327.s003.docx]

**
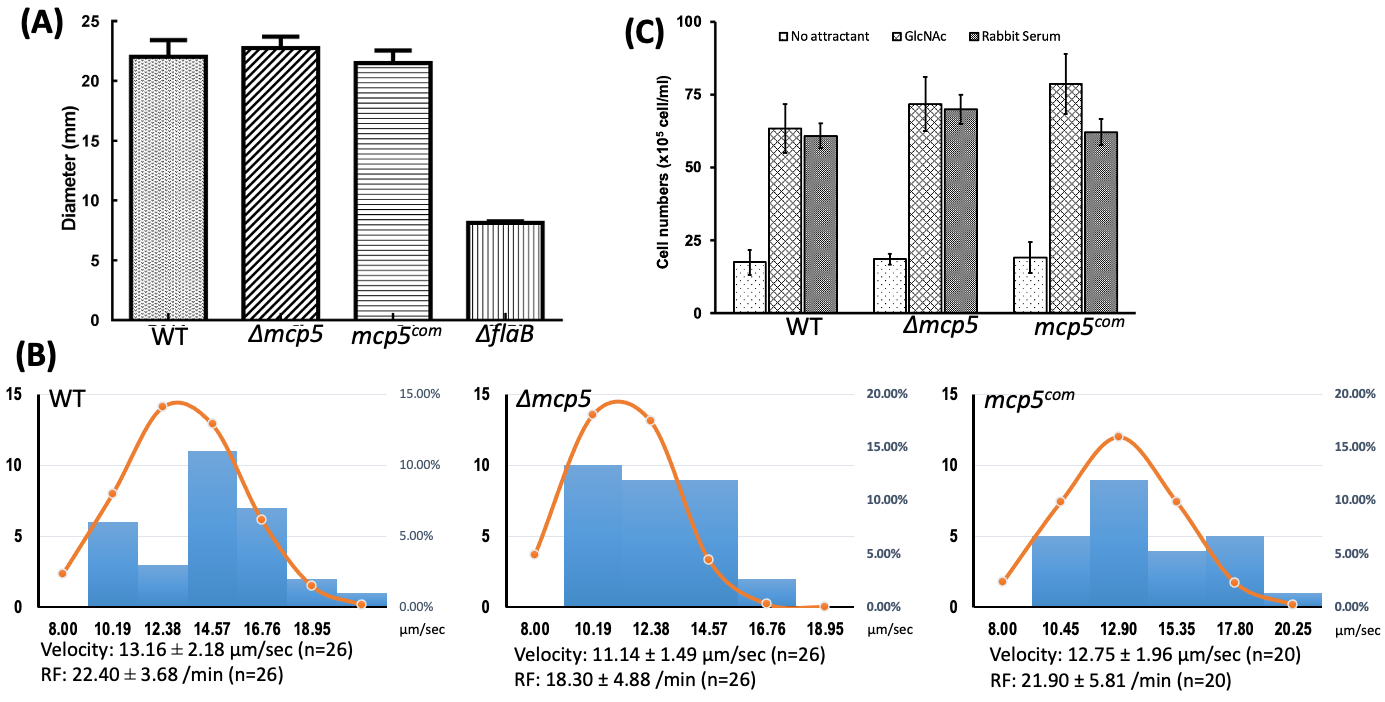
**

**S1_Fig. The *mcp5* mutant has no defect in motility and chemotaxis *in vitro.*** (**A**) Swimming plate assay. Swimming plate assays for wild-type *B. burgdorferi* B31M, the *mcp5* mutant, and the *mcp5* complemented strain were performed using 0.35% agarose with BSK-II medium diluted 1:10 with DPBS. The diameters of the swim rings were measured, and average diameters of each strain were calculated from four independent plates. The *flaB* mutant (Δ*flaB*) served as a negative control. (**B**) Motion tracking analysis. Spirochetes were video captured using a computer-based motion tracking system. The average cell swimming velocities (μm/s) of tracked cells were calculated. (**C**) Capillary chemotaxis assays. *B. burgdorferi* cells were resuspended in the motility buffer and subjected to capillary assays for chemotaxis to acetylglucosamine and rabbit serum. Spirochetes were enumerated using Petroff-Hausser counting chambers under a dark-field microscope. For the tracking, swimming plate, and capillary assays, the results are expressed as means ± standard errors of the means (SEM). The significance of the difference between different strains was evaluated with an unpaired Student *t* test (*P* value < 0.01).
