# Supplementary material for: MCP5, a methyl-accepting chemotaxis protein regulated by both the Hk1-Rrp1 and Rrp2-RpoN-RpoS pathways, is required for the immune evasion of Borrelia burgdorferi
Source: PLoS Pathog. 2024 Dec 30;20(12):e1012327. doi: 10.1371/journal.ppat.1012327 (PMC11723614; doi:10.1371/journal.ppat.1012327)
Supplement: S2 Fig — Spleens from C3H/HeN mice of untreated or treated with anti-Asiola-GM1 blocking antibody were harvested, and single-cell suspensions were prepared as outlined in the Materials and Methods. Cells were stained with antibodies against CD45, CD3, and CD49b. The gating strategy shown identifies NK cell populations (CD45+CD3-CD49b+) by excluding debris, focusing on single, live cells. Single-stained and unstained controls were used to define gating parameters and ensure accurate compensation. (DOCX) [file ppat.1012327.s004.docx]

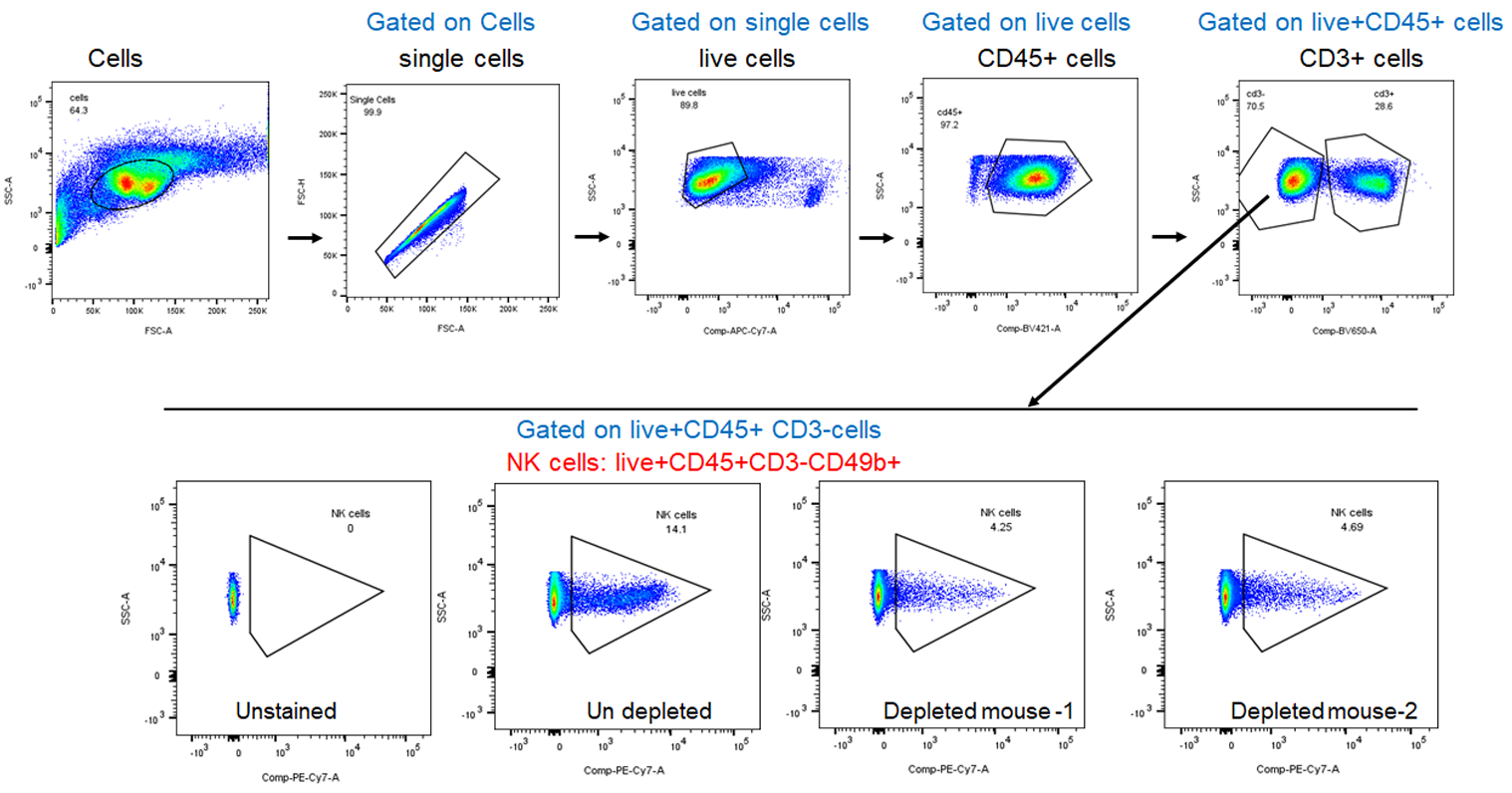


**S2_Fig. Representative gating strategy to assess NK cell depletion in C3H mice.** Spleens from C3H/HeN mice of untreated or treated with anti-Asiola-GM1 blocking antibody were harvested, and single-cell suspensions were prepared as outlined in the Materials and Methods. Cells were stained with antibodies against CD45, CD3, and CD49b. The gating strategy shown identifies NK cell populations (CD45+CD3-CD49b+) by excluding debris, focusing on single, live cells. Single-stained and unstained controls were used to define gating parameters and ensure accurate compensation.
