# Supplementary material for: MCP5, a methyl-accepting chemotaxis protein regulated by both the Hk1-Rrp1 and Rrp2-RpoN-RpoS pathways, is required for the immune evasion of Borrelia burgdorferi
Source: PLoS Pathog. 2024 Dec 30;20(12):e1012327. doi: 10.1371/journal.ppat.1012327 (PMC11723614; doi:10.1371/journal.ppat.1012327)
Supplement: S3 Fig — Spleens from SCID mice of untreated or treated with anti-Asiola-GM1 blocking antibody were harvested, and single-cell suspensions were prepared as outlined in the Materials and Methods. Cells were stained with antibodies against CD45, CD3, and CD49b. The gating strategy shown identifies NK cell populations (CD45+CD3-CD49b+) by excluding debris, focusing on single, live cells. Single-stained and unstained controls were used to define gating parameters and ensure accurate compensation. (DOCX) [file ppat.1012327.s005.docx]

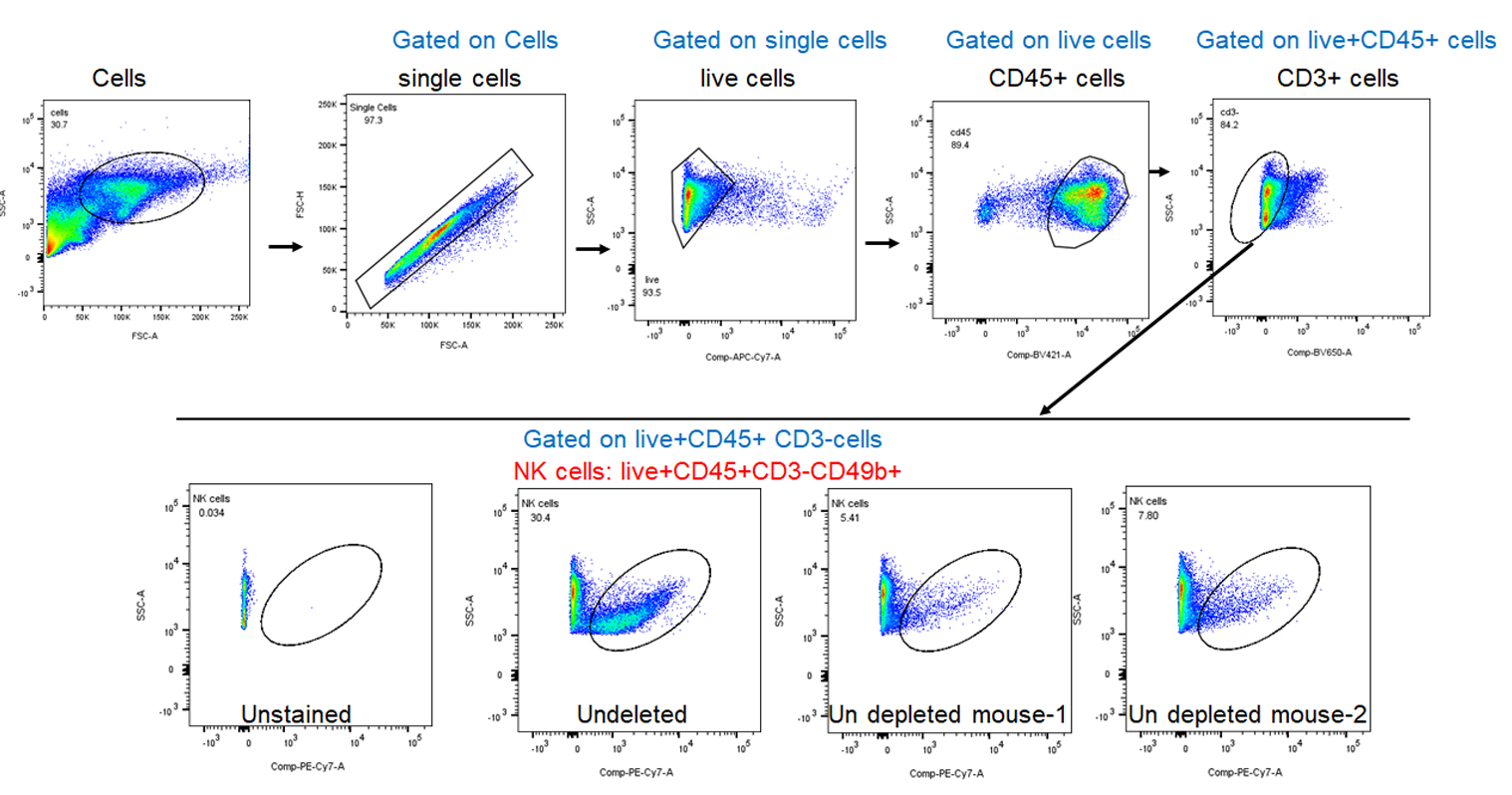


**S3_Fig. Representative gating strategy to assess NK cell depletion in SCID mice.** Spleens from SCID mice of untreated or treated with anti-Asiola-GM1 blocking antibody were harvested, and single-cell suspensions were prepared as outlined in the Materials and Methods. Cells were stained with antibodies against CD45, CD3, and CD49b. The gating strategy shown identifies NK cell populations (CD45+CD3-CD49b+) by excluding debris, focusing on single, live cells. Single-stained and unstained controls were used to define gating parameters and ensure accurate compensation.


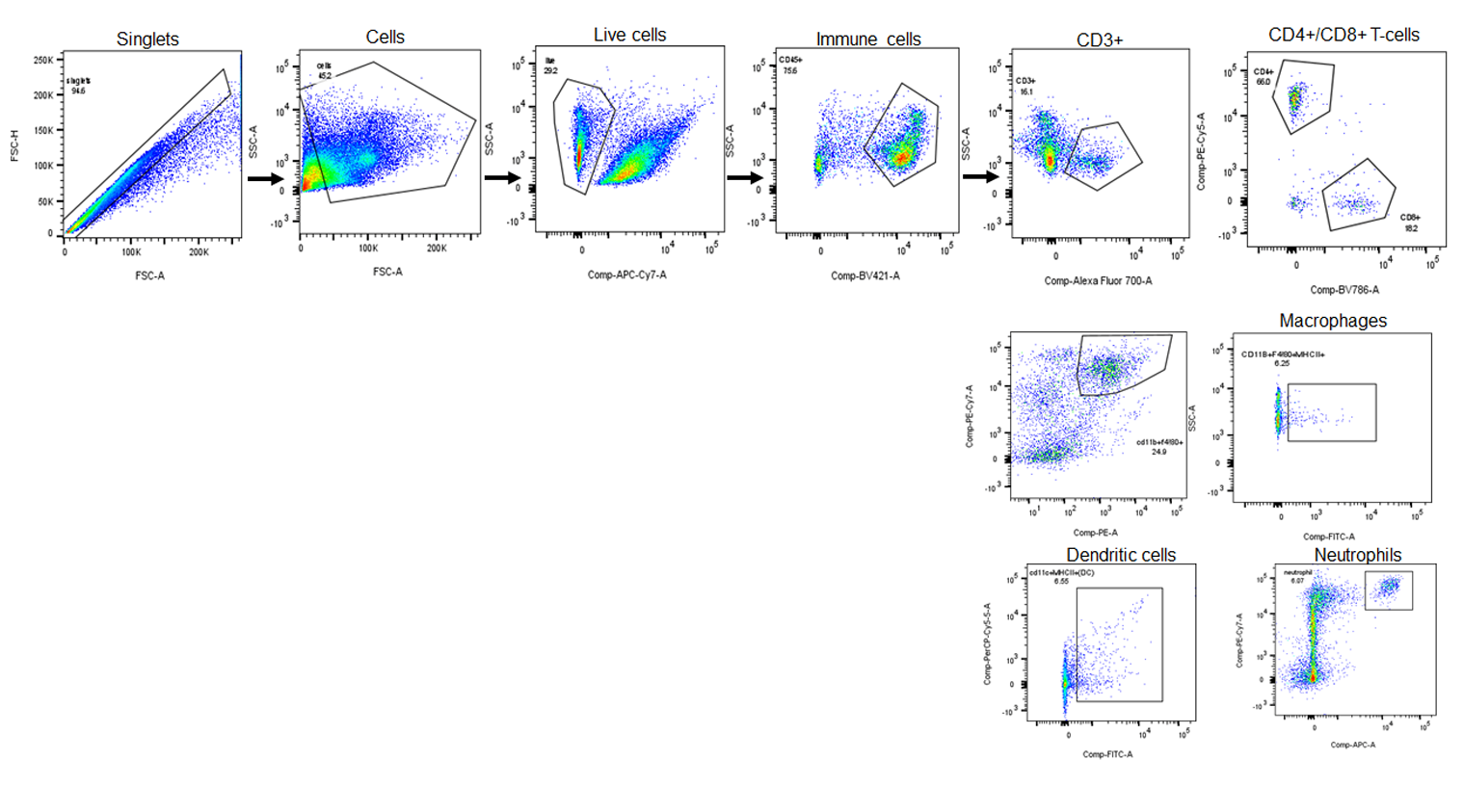


**Fig. S4. Gating strategy to assess immune cell infiltration at the site of infection.** Skin cells from wild-type or NK cell-depleted C3H mice (n=4) challenged with the mcp5 mutant (1 × 10^5/mouse) were harvested and dissociated as described in Materials and Methods. Cells were stained with antibodies against CD45, CD3, CD4, CD8a, CD11b, F4/80, CD11c, and I-A/I-E. The outlined gating strategy shows the approach used to identify the following populations, excluding debris and gating on single cells: live cells, CD45+ cells, CD45+CD3+CD4+ T cells, CD45+CD3+CD8+ T cells, CD45+CD11b+F4/80+I-A/I-E+ macrophages, CD45+CD11c+I-A/I-E+ dendritic cells, and CD45+CD11b+Ly6G+ neutrophils. Single-stained and unstained controls were used to set gating parameters and ensure proper compensation.
