# Supplementary material for: MCP5, a methyl-accepting chemotaxis protein regulated by both the Hk1-Rrp1 and Rrp2-RpoN-RpoS pathways, is required for the immune evasion of Borrelia burgdorferi
Source: PLoS Pathog. 2024 Dec 30;20(12):e1012327. doi: 10.1371/journal.ppat.1012327 (PMC11723614; doi:10.1371/journal.ppat.1012327)
Supplement: S4 Fig — Skin cells from wild-type or NK cell-depleted C3H mice (n = 4) challenged with the mcp5 mutant (1 × 10^5/mouse) were harvested and dissociated as described in Materials and Methods. Cells were stained with antibodies against CD45, CD3, CD4, CD8a, CD11b, F4/80, CD11c, and I-A/I-E. The outlined gating strategy shows the approach used to identify the following populations, excluding debris and gating on single cells: live cells, CD45+ cells, CD45+CD3+CD4+ T cells, CD45+CD3+CD8+ T cells, CD45+CD11b+F4/80+I-A/I-E+ macrophages, CD45+CD11c+I-A/I-E+ dendritic cells, and CD45+CD11b+Ly6G+ neutrophils. Single-stained and unstained controls were used to set gating parameters and ensure proper compensation. (DOCX) [file ppat.1012327.s006.docx]

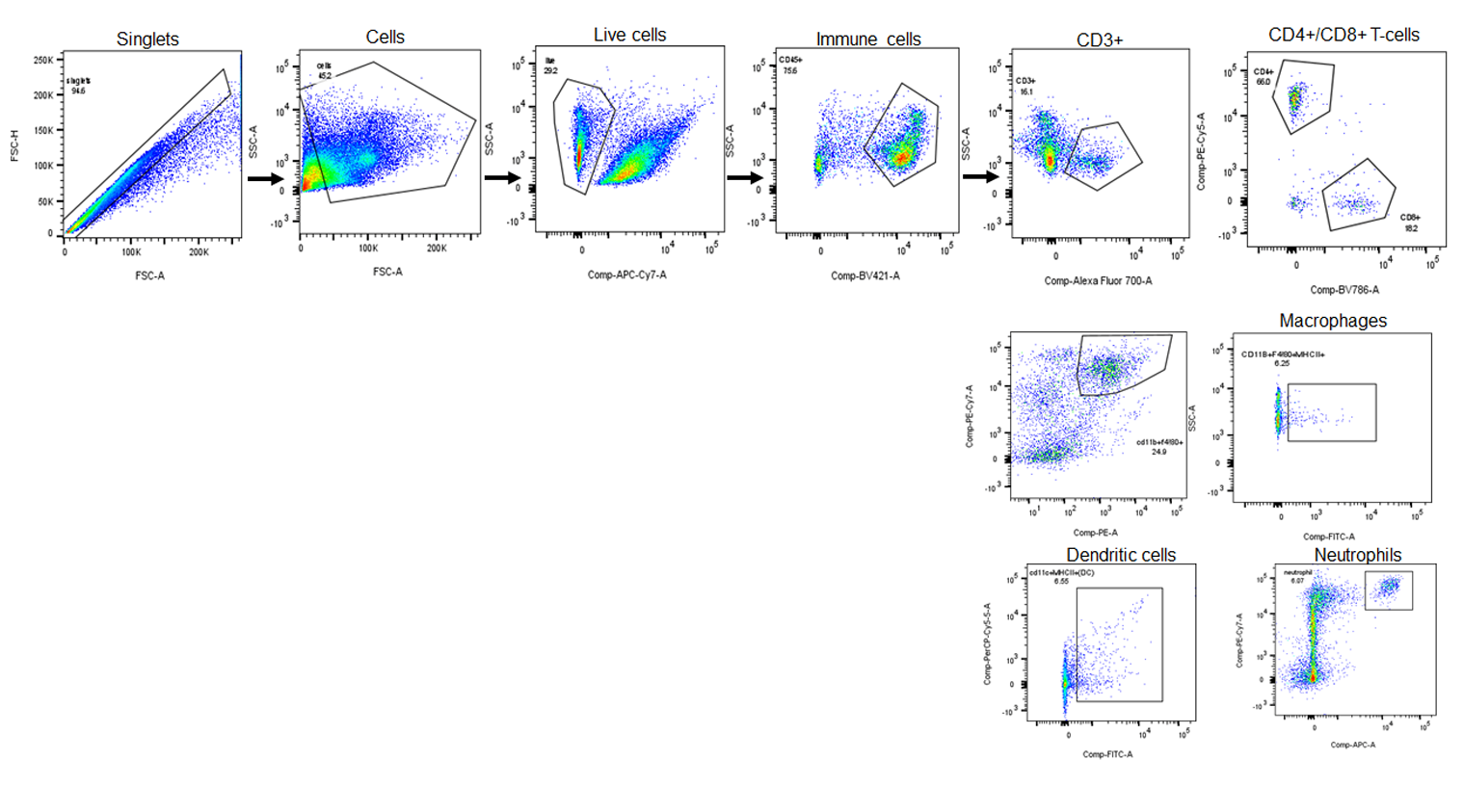


**S4_Fig. Gating strategy to assess immune cell infiltration at the site of infection.** Skin cells from wild-type or NK cell-depleted C3H mice (n=4) challenged with the mcp5 mutant (1 × 10^5/mouse) were harvested and dissociated as described in Materials and Methods. Cells were stained with antibodies against CD45, CD3, CD4, CD8a, CD11b, F4/80, CD11c, and I-A/I-E. The outlined gating strategy shows the approach used to identify the following populations, excluding debris and gating on single cells: live cells, CD45+ cells, CD45+CD3+CD4+ T cells, CD45+CD3+CD8+ T cells, CD45+CD11b+F4/80+I-A/I-E+ macrophages, CD45+CD11c+I-A/I-E+ dendritic cells, and CD45+CD11b+Ly6G+ neutrophils. Single-stained and unstained controls were used to set gating parameters and ensure proper compensation.
